# Spontaneous Crimping of Gelatin Methacryloyl Nanofibrils Induced by Limited Hydration

**DOI:** 10.1021/acsbiomaterials.5c00828

**Published:** 2025-07-18

**Authors:** Chien-Wei Wu, Tzu-Yin Huang, Liang-Jie Huang, Yi-Wei Kuo, Chin-Lin Guo, Po-Ling Kuo

**Affiliations:** † Graduate Institute of Biomedical Electronics and Bioinformatics, 33561 National Taiwan University, No. 1, Sec. 4, Roosevelt Rd., Taipei 106, Taiwan; ‡ Department of Electrical Engineering, National Taiwan University, No. 1, Sec. 4, Roosevelt Rd., Taipei 106, Taiwan; § Institute of Physics, 38017 Academia Sinica, No. 128, Academia Rd. Sec. 2, Taipei 115, Taiwan; ∥ Department of Physical Medicine and Rehabilitation, National Taiwan University Hospital, No.1, Chang De St., Taipei 100, Taiwan; ⊥ College of Medicine, National Taiwan University, No.1, Jen Ai Rd. Sec. 1, Taipei 100, Taiwan

**Keywords:** Collagen derivatives, 3D cell culture, fibril crimping, GelMA, tissue scaffold

## Abstract

The crimped appearance of native collagen fibrils in youthful tissues serves as a mechanical buffer and phenotype determinant for resident cells. *In vitro* platforms emulating these native crimped networks facilitate the study of cell-matrix dynamics in various pathophysiological contexts. However, creating fibrillar networks with sizes and crimping matching native tissues using collagen-derived substrates remains challenging. We present an innovative approach to produce spontaneous, tunable crimping of electrospun, aligned gelatin methacryloyl nanofibrils using limited hydration. The diameter of the synthesized fibrils approximated that of native fibrils. Beyond individual fibril crimping, the network exhibited large-scale, periodic crimping with wavelengths matching native collagen networks. Tensile stress tests revealed that crimping reduced network stiffness but enhanced stretchability, consistent with native tissues. Additionally, crimping promoted cell translocation into the network. Fibroblasts cultured on crimped fibrils showed smaller cell areas, higher vinculin and α-tubulin expression, and lower α-smooth muscle actin levels compared to those on straight fibrils. This novel method not only replicates the native fibril characteristics using collagen-derived materials, but also offers a valuable tool for advancing our understanding of cell-matrix interactions, with significant implications for tissue engineering and regenerative medicine.

## Graphical abstract

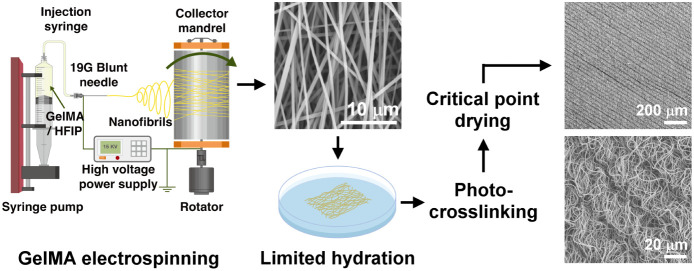

## Introduction

The collagen fibrillar network, the most prevalent fundamental structure in the body, typically exhibits a wavy or “crimped” morphology across various tissues,^1,2^ encompassing tendons,^3,4^ ligaments,^5^ joints,^6^ arterial walls,^7,8^ intervertebral discs,^9^ intestines,^10^ peripheral nerve,^11^ cornea, and sclera.^12^ Depending on age, species, and tissue types, the fibril diameter ranges from 30 to 300 nm,^1,13,14^ while the crimp wavelength, measured as the distance between adjacent peaks in the crimp waveform, ranges from 10 to 100 μm.^14,15^ This crimped pattern is more prominent in youthful tissues but diminishes with age^3,12,16,17^ and tissue damage.^18^ The initial 1–4% of network stretching primarily results from the straightening of these crimped structures,^3,19,20^ with reduced crimping associated with increased network stiffness.^8^ This renders the crimped pattern as a mechanical buffer to protect resident cells from stress during stretching,^17,20,21^ as seen in peripheral nerves, where the crimped collagen fibrils surrounding the bundled axons in the endoneurium shield the axons from tensile forces, allowing them to remain relaxed during body movement.^11^

Several methods have been developed to create wavy or crimped nanofibrils with wavelengths at the microscale, using synthetic polymers. Varesano et al. utilized air flow to curl up electrospun polyamide-6 (PA6) nanofibrils into a wavy structure with an averaged wavelength of 10 μm.^22^ Liu et al. induced waviness in electrospun poly lactic acid (PLA) nanofibrils using ethanol, achieving wavelengths around 5–100 μm.^21^ Magnetic fields applied to electrospun jets of poly­(lactic-*co*-glycolic) acid (PLGA) or polyvinylpyrrolidone (PVP) also produced wavy nanofibrils.^23^ Surrao et al.^24^ and Chao et al.^25^ fabricated wavy poly­(l-lactide-*co*-ε-caprolactone) (P­(LLA-CL)) and poly-l-lactic acid (PLLA) nanofibrils with wavelengths around 15–55 μm by transiently heating the electrospun nanofibrils over their glass-transition temperatures. Davidson et al. induced wavy morphology on electrospun dextran vinyl sulfone (DexVS) fibrils by swelling them between two anchored ends.^26^ Through these techniques, crimped structures have been shown to influence morphology and behavior of resident cells. For example, the morphology of fibroblasts and stem cells cultured on PLLA and PLGA nanofibrils, respectively, conformed to the synthetic crimped patterns, similar to the cellular shapes observed in fibroblasts embedded in the natural crimped patterns of chicken tendons.^23,25,27^ Fibroblasts cultured on wavy PLA nanofibrils exhibited enhanced strain tolerance, as the unfolding of the crimped structure under applied stress directly reduced the mechanical strain experienced by the cells.^21^ Human umbilical vein endothelial cells cultivated within wavy DexVS fibrils displayed decrease in cell spread area, nuclear area, focal adhesion area, migration speed, and nuclear to cytoplasmic ratio of Yes-associated protein (YAP).^26^

However, these studies have largely relied on synthetic, nonproteinaceous materials that cannot fully recapitulate the biological functionalities of collagen. To better understand the impact of naturally occurring crimped fibrils on cell morphology and phenotype, developing three-dimensional (3D) cell culture platforms with collagen-derived crimped fibrils is essential. These platforms should feature adjustable crimped patterns with wavelengths of 10–100 μm and nanoscale diameters to mimic those found in native tissues under various pathophysiological conditions, thereby facilitating the exploration of interactions between resident cells and crimped structures. Despite advancements in creating crimped nanofibrils using synthetic polymers, challenges remain in synthesizing similar structures from collagen-derived materials. For instance, Caves et al. sandwiched collagen solution between a flat and a microridged polyurethane membrane that was both prestrained, producing crimped collagen fibers with wavelengths of hundreds of microns upon releasing the strain.^28^ They also employed a MEMS-based micromolding approach to generate crimpled collagen fibers with similar wavelength.^29^ However, the yielded fibril diameters were tens of microns, exceeding those typically found in natural tissues.

Here, we present a novel and straightforward approach to create crimped nanofibrils from gelatin methacryloyl (GelMA) for 3D cell culture and tissue engineering. GelMA, a cost-effective, methacrylate-modified gelatin derivative, is widely used in tissue engineering due to its versatility.^30^ As a collagen derivative, gelatin provides biological cues for cellular adhesion and matrix remodeling, whereas the methacrylate component enhances mechanical and thermal stability of the construct through photo-cross-linking.^31^ We synthesized fibrillar networks of GelMA via electrospinning, observing spontaneous, controllable crimping with limited hydration. Our exploration of variations in fibril topology reveals their significant effects on both the mechanical properties of the networks and the phenotypes of cultured cells. By demonstrating crimped nanofibrils derived from proteinaceous materials, our work addresses a critical gap in the field, laying the foundation for understanding and leveraging the interplay between fibril-crimped topology and cellular behavior.

## Materials and Methods

### Synthesis of Lyophilized GelMA

GelMA was synthesized by replacing the amino groups of gelatin with methacryloyl groups using methacrylate anhydride (MA) as previously described^32,33^ (Figure 1a). A 10%(w/v) gelatin (SI-G2500- 500G Gelatin 300, Type A, Sigma-Aldrich, St. Louis, MO, USA) solution was prepared by thoroughly dissolving 80 g of gelatin in 800 mL of 0.1 M carbonate–bicarbonate (CB) buffer (BupHTM Carbonate-Bicarbonate Buffer Packs, Thermo Fisher Scientific, Waltham, MA, USA) with stirring at 240 rpm at 80 °C. MA (Sigma-Aldrich) was added in six increments of 1.66 mL, with 30 min intervals, to achieve a MA to gelatin ratio of 1 mL/10 g,^32,34^ while adjusting the pH to 9.00 using 2 M NaOH solution after each addition. The mixture was stirred overnight and then pH-adjusted to 7.40. The resulting GelMA solution was enclosed in dialysis membranes (12–14 kDa Standard RC Membrane, Repligen, Waltham, MA, USA) and submerged in distilled water at 45 °C for 4 days to remove unreacted methacrylic anhydride and byproducts such as methacrylic acid. This extensive purification step, adapted from standard protocols,^32,34^ is critical for minimizing the presence of residual species that could otherwise interfere with the reproducibility or interpretation of cross-linking behavior. The distilled water was sequentially refreshed at intervals of 6, 6, 12, 12, 12, 24, and 24 h. The pH value was adjusted to 7.40 at the end of dialysis. The solution was frozen at −80 °C on aluminum plates, lyophilized by a freeze-dryer (SFD-25, Evergreen, Taipei, Taiwan), and stored at −20 °C.

**Figure 1:**
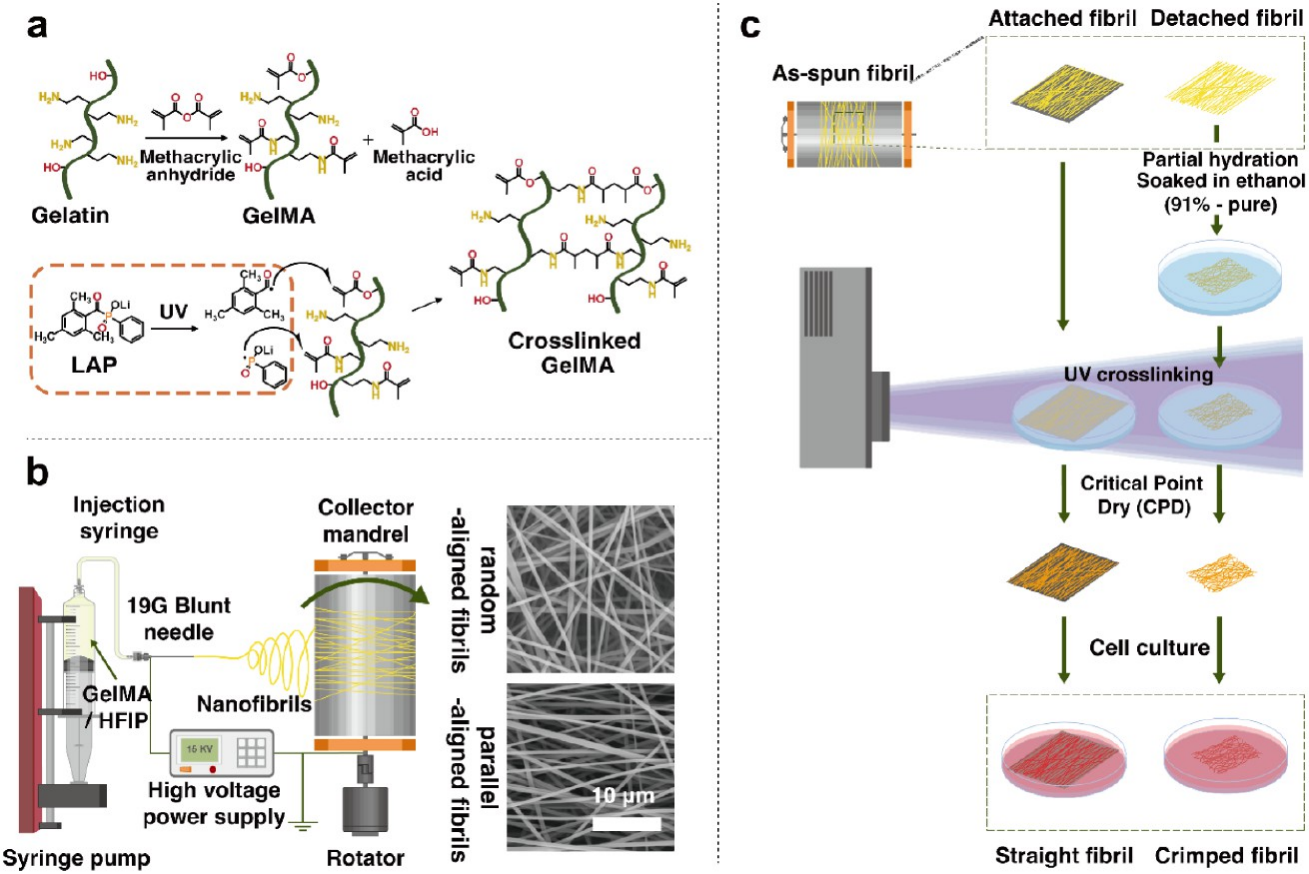
Schematic diagrams for synthesis of crimped GelMA fibrils. (a) Structural formula representation for GelMA synthesis and photo-cross-linking. (b) Setup for generating random and parallel-aligned GelMA fibrils using electrospinning. (c) Procedure to induce crimping in the electrospun fibrils. To produce crimped GelMA fibrils, the fibril network was detached from the aluminum foil, soaked in 91%-pure ethanol solutions, photo-cross-linked, and dried using CPD for subsequent cell culture. Fibril networks that remained attached to the foil and were treated with pure ethanol solution maintained a straight morphology.

### ^1^H NMR/TNBS Analysis for MA Substitution

The degree of MA substitution in the synthesized GelMA was determined by ^1^H NMR and TNBR analysis. For the ^1^H NMR analysis, one gelatin and four freeze-dried GelMA samples were cut into small pieces, and 10–15 mg of the yielded powders were dissolved in 600 μL of 99.9 atom % deuterium oxide (Sigma-Aldrich) in a 5 mm NMR tube (Wilmad-LabGlass, Vineland, NJ, USA). The ^1^H NMR spectra were recorded using a solution-state NMR (Bruker AVII-500, Billerica, MA, USA) at room temperature. The degree of substitution (DS_NMR_, %) was calculated by dividing the lysine methylene proton value of synthesized GelMA macromers by the corresponding value in type-A gelatin. The lysine methylene proton value was determined from the integral area of the ^1^H NMR spectra at 2.85–2.95 ppm and processed using MestReNova software (Mestrelab Research, Santiago de Compostela, Spain). To quantify the amino groups in the synthesized GelMA using the TNBS assay, one gelatin and four GelMA samples were separately dissolved at 1.6 mg/mL in a 0.1 M sodium bicarbonate buffer (ECHO CHEMICAL, Taichung City, Taiwan). Each 0.5 mL sample was mixed with 0.5 mL of 0.1% TNBS (Sigma-Aldrich) and incubated at 37 °C for 2 h, followed by adding 0.25 mL of 1 M hydrogen chloride (ECHO CHEMICAL) and 0.5 mL of 10% sodium dodecyl sulfate (Sigma-Aldrich) to stop the reaction. Absorbance at 335 nm was measured to detect the number of amino groups. The TNBS degree of substitution (DS_TNBS_, %) was calculated by dividing the absorbance of GelMA by that of gelatin.

### Preparation of Electrospun GelMA Fibrils

GelMA solutions of various concentrations (6%, 7%, 8%, 9%, 10%, 11%, 12% w/v) were prepared by dissolving lyophilized GelMA in HFIP (1,1,1,3,3,3-hexafluoro-2-propanol, Sigma-Aldrich). The solutions were filtered through a 0.45 μm PTFE syringe filter (Labfil, ALWSCI Corporation, Zhejiang, China), and put into a 5 mL syringe connected to a syringe pump and attached with a 19 G blunt tip needle. The collectors, either plate or spinning mandrel, were covered with aluminum foil and placed 15 cm from the needle tip. A cross voltage of 15 kV was applied between the source and collector, and the GelMA solution was pumped at a rate of 1.0 mL/h. The spinning mandrel collector (radius = 5 cm, length = 15.5 cm) rotated at 1600 rpm. After 3 h of electrospinning, the GelMA fibril network was removed from the collector and stored on aluminum foil at room temperature (Figure 1b). To induce a crimped morphology, the fibril network was soaked in ethanol solutions of varying concentrations (91%, 93%, 95%, 97%, pure) for different durations. Photo-cross-linking was performed by immersing the networks in a 0.25% (w/v) LAP (lithium phenyl-2,4,6-trimethyl-benzoyl phosphinate, LAP. 900889, Sigma-Aldrich) solution prepared in 95% ethanol protected from light, followed by exposure of 365 nm UV light (UV projector, H6510BD, Acer, Taipei, Taiwan) at a distance of 12 cm for 25 min, with a UV light power of 0.344 mW/cm^2^, measured by a UVX Radiometer (AG, Analytik Jena, Jena, Germany) and a UVX-36 Longwave sensor (Artisan Technology Group, Champaign, Illinois, USA) (Figure 1c).

### Critical Point Drying for Fibril Networks

The photo-cross-linked GelMA fibril networks were washed three times with 95% ethanol to remove excessive photoinitiator (LAP), followed by an additional 20 min soak in 95% ethanol. LAP is soluble in both water and ethanol, and this washing protocol ensures effective removal of residual initiator. SEM images demonstrate that no detectable amount of LAP remains in the final washed materials (Figure S1). The samples were dehydrated by replacing the 95% ethanol with absolute ethanol, stored in the latter for at least 20 min, and transferred into the CPD specimen baskets associated with a CPD machine (HCP-2 Critical Point Dryer, Hitachi, Tokyo, Japan). The CPD process was performed by increasing the temperature to approximately 35 °C and the pressure to about 1,200 psi to reach the critical point of CO_2_, where liquid CO_2_ transitions to vapor without a change in density, preventing surface tension effects that could damage the nanostructure of the fibrils. The absolute ethanol acts as an intermediate fluid, being miscible with both liquid CO_2_ and water, facilitating the removal of water from the fibrils.

### Morphology Measurement for GelMA Fibrils: Fibril Diameter, Alignment, Crimping Degrees, and Crimping Wavelength

The dried fibril networks were mounted on a scanning electron microscope (SEM) specimen stub using conductive tape, sputter-coated with gold (VD MSP-1S Magnetron, Vacuum Device Inc., Ibaraki, Japan) for 90 s, and scanned with an SEM (Hitachi TM3000, Hitachi, Tokyo, Japan) at an accelerating voltage of 15 kV. The fibril diameter, alignment, and crimping degree were determined and analyzed by ImageJ.^46^ Alignment was determined by calculating the angle of each fibril relative to the mean fibril angle. The crimping degree was calculated as the ratio of the straight distance between the end points of the fibril to its contour length. All measurements were based on 50 fibrils. In addition to the wavy morphology of individual fibrils, the network exhibited a coherent, large-scale crimping pattern when observed at a larger scale. To measure the wavelength of this large-scaled crimping (the distance between adjacent crimp valleys), we applied several image processing techniques: histogram equalization to enhance contrast, image blurring to reduce background noise and smooth edges, the Canny edge detection algorithm to identify crimp edges, and the Hough Line transformation to fit these edges as straight lines. The wavelengths were then determined by averaging the distances between adjacent lines.

### Swelling Ratio Measurement for Fibril Networks

Both GelMA hydrogels and electrospun fibril networks were photo-cross-linked, dried using the CPD process, weighted, soaked in deionized water, and repeatedly weighed at specific intervals to determine the wetted weight of the materials. The swelling ratio of a sample was calculated as the difference between the wetted and dried weights, divided by the dry weight.

### Tensile Measurements for Fibril Networks

Tensile measurements were conducted using a mechanical test instrument (ElectroForce 3100, TA Instruments, New Castle, DE, USA). The samples used for mechanical testing were prepared by cutting dried electrospun mats into dimensions of 10 mm × 5 mm × 0.25 mm, clamped, and stretched along the principal axis of fibril alignment. The lateral dimensions were measured using an absolute digimatic caliper (Mitutoyo, 500-173-30, Kanagawa, Japan). The sample thickness was controlled by maintaining a fixed electrospinning duration of 3 h and subsequently verified using a noncontact laser displacement sensor (optoNCDT1420; Micro-Epsilon, Taipei, Taiwan). Stress–strain curves were recorded at a stretching speed of 0.05 mm/s until mechanical failure of the samples. For the wetted groups, samples were soaked in phosphate buffer (PBS) for 2 h prior to testing.

### Cell Types and Cell Culturing

The cell types used were NIH-3T3 fibroblasts (Bioresource Collection and Research Centre (BCRC), Hsinchu, Taiwan) and bone marrow-derived mesenchymal stem cells (BMSCs) from normal human sources (ATCC PCS-500–012TM, Manassas, VA, USA). NIH-3T3 cells were cultured in DMEM (High Glucose, Simply, GeneDireX Inc., Taoyuan, Taiwan) supplemented with 10% fetal bovine serum (FBS, Gibco, Thermo Fisher Scientific) and 1% Penicillin/Streptomycin (ABL02, Caisson, Smithfield, UA, USA). BMSCs were cultures in Mesenchymal Stem Cell Basal Medium (ATCC PCS-500-030) supplemented with Mesenchymal Stem Cell Growth Kit (ATCC PCS-500-041) and 1% Penicillin/Streptomycin (ABL02, Caisson). Both cell types were incubated at 37 °C with 5% CO_2_. Adherent cells were detached using trypsin after reaching over 80% confluency and seeded onto a dried GelMA fibril network placed in a Petri dish at a density of 10^4^ cells/cm^2^.

### Cell Viability Test

Cell viability was determined using 1 μL/mL Calcein AM (494/517 nm, BioVision, Abcam Scientific, Bristol, UK) and 2 μL/mL Propidium Iodide (535/617 nm, BioVision) staining at 0, 24, and 48 h postseeding. After a 30 min incubation period, fluorescence images were captured by an inverted fluorescence microscope (IX71, Olympus, Shinjuku, Tokyo, Japan) equipped with a 10× objective lens (numerical aperture = 0.25, Olympus, Shinjuku, Tokyo, Japan). For cells cultured for 48 h, viability images were also acquired using confocal microscopes (TCS SP5 or SP8 Confocal Spectral Microscope Imaging System, Leica, Wetzlar, Germany) equipped with 10× objective lens. Calcein AM and Propidium Iodide were excited with light at 490 and 545 nm, respectively. The number of live and dead cells was quantified using ImageJ, and cell viability was calculated by dividing the count of live cells by the total cell count.

### Cell SEM Imaging

To observe cell morphology on GelMA fibril networks using SEM, the networks with the cells cultured for 24 h were washed with 0.1 M PBS to remove the medium and soluble proteins, soaked in a PBS solution containing 2.5% glutaraldehyde for 1 h to fix the cells, and washed with dd-water three times to remove the fixative solution. The samples were dehydrated by sequentially soaking in ethanol solutions of increasing concentrations (35%, 50%, 65%, 75%, 85%, 95%, and pure) for 30 min each. The samples underwent the CPD process and were then observed via SEM.

### Cell Immunofluorescence Staining

To observe the cytoskeleton and cell nucleus, the samples were transferred to a confocal dish, washed three times with PBS, fixed by 4% paraformaldehyde (ChemCrus, Santa Cruz Biotechnology, Dallas, TX, USA) in PBS for 15 min, washed again with PBS, permeabilized by 1% triton X-100 (J.T. Baker, Avantor, Radnor, PA, USA) for 10 min, and blocked in 2% BSA (Bovine Serum Albumin, Sigma-Aldrich) for 2 h. Monoclonal primary antibodies against vinculin (1:500 in PBS/BSA, V9264 #0000152189, antivinculin antibody, mouse monoclonal, Sigma-Aldrich), alpha-tubulin (1:500 in PBS/BSA, antialpha-tubulin antibody, AB18251 #1043566-5, rabbit monoclonal, Sigma-Aldrich), α-smooth muscle actin (α-SMA, 1:500 in PBS/BSA, 1A4 14-9760-82 #2702005, mouse monoclonal, eBioscience, Invitrogen) and YAP1 (1:500 in PBS/BSA, SU33-06 MA5-32117 #YJ4102655, recombinant rabbit monoclonal, Invitrogen) were added and incubated overnight at 37 °C. After washing with PBS, secondary antibodies (1:500 polyclonal Alexa Fluor-488 goat antimouse, Alexa Fluor-561 goat antirabbit, Invitrogen, Thermo Fisher Scientific) were added and incubated for 3 h at room temperature, protected from lights, followed by another PBS washing. Samples were then stained with phalloidin (Alexa Fluor 633 phalloidin, #2604076, Invitrogen, Thermo Fisher Scientific) against filamentous actin (F-actin) for 1 h and DAPI (DAPI solution, #2743087, Thermo Fisher Scientific) against DNAs for 15 min. The stained cells were observed using a TCS SP5 confocal spectral microscope imaging system (Leica) equipped with 40× and 100× objectives. The fluorescence images were acquired using the same setup to facilitate data comparison. To quantify fluorescence intensity, we first segmented cells stained for F-actin and nuclei using Fiji software with the deep learning-based TrackMate-Cellpose plugin. Fluorescence intensities for vinculin, tubulin, and α-SMA were determined on a per-cell basis by dividing the total fluorescence signal of each respective protein within the delineated cell area by the cell area. The YAP1 nuclear-to-cytoplasmic ratio was calculated for each cell by dividing the total fluorescence intensity in the nucleus by the fluorescence intensity in the cytoplasm.

### Cell Cytotoxicity Evaluation

We used Cell Counting Kit-8 (MedChemExpress, Monmouth Junction, NJ, USA) to analyze the cytotoxicity of the fibril mats. For the experimental group (Group Crimped Fibril), the medium was prepared by soaking 1 cm × 1 cm fibril mat in DMEM for 24 h. The negative control group (Group Blank, 100% live cells) used the cell culture medium, while the positive control (Group Bleach, 0% live cells) used a mixture of bleach and cell culture medium at a 1:100 ratio. NIH-3T3 cells were seeded in a 96-well plate at a concentration of 10^4^ cells per well and incubated at 37 °C with 5% CO_2_ for 24 h to allow cell adhesion. The culture medium was then replaced with the prepared solutions for each of the three groups, and the cells were cultured for an additional 24 h. Each group had five replicates. After the incubation, the culture medium in each well was replaced by a mixture of 100 μL DMEM and 10 μL CCK8 reagent, followed by 2 h of incubation. Optical density (OD) at 450 nm was measured using a microplate reader (SpectraMax iD3, KIMFOREST, Montebello, CA, USA). Cell viability was determined by dividing the OD values of each group by that of the negative control.

### Cell Aspect Ratio Calculation

The aspect ratio of cells cultured on fibril networks, defined as the ratio of the major to minor axis lengths, was calculated from fluorescence images stained for F-actin. An aspect ratio of 1 indicated that the cell was round-shaped, while larger values indicated an elongated morphology. The aspect ratios of over 50 cells were calculated for each image.

### Statistical Analysis

Statistical analyses began with the Shapiro-Wilk test to assess data normality. If the data were normally distributed, Levene’s test was performed to evaluate variance equality. For data with equal variances, one-way or two-way ANOVA, followed by Tukey’s HSD post hoc test, were used for multivariate analysis, and unpaired or paired *t*-tests for bivariate analysis. For data with unequal variances, Welch’s or Brown-Forsythe tests were used for multivariate analysis, followed by the Games–Howell post hoc test. Welch’s *t*-test was used for bivariate analysis. Nonnormally distributed data were analyzed using the Kruskal–Wallis test with Dunn’s post hoc test for multivariate analysis. Mann–Whitney U tests and Wilcoxon signed-rank tests were used for unpaired and paired bivariate analyses, respectively. Pairwise Levene’s tests compared variances in multivariate or nonnormal bivariate analyses, while Fisher’s *F*-tests were used for normal bivariate analysis. Statistical significance was set at *p* < 0.05. Outliers, defined as values beyond 1.5 times the interquartile range (IQR) from the first or third quartile, were identified and marked on box plots. Normal curves overlaid on histograms matching the mean, standard deviation, and area under the curves to those of the histograms were calculated.

## Results

### Spontaneous GelMA Network Shrinkage and Fibril Crimping Upon Limited Hydration

GelMA was synthesized by grafting methacrylate groups onto the gelatin backbone via reactions between methacrylic anhydride and lysine residues, as previously described^32,33^ (Figure 1a). The degree of MA substitution in the synthesized GelMA averaged 94.14% and 79.31%, as measured by ^1^H NMR and TNBS analysis, respectively (Figure S2 and Table S1). The lower value from the TNBS assay is attributed to its tendency to underestimate the degree of substitution.^35^ Electrospun GelMA fibrils were generated using 6–12% GelMA solutions and collected on aluminum foils (Figure 1b). The identical characteristic peaks at 5.4–5.5 ppm and 5.6–5.7 ppm in the ^1^H NMR spectra of both GelMA and the electrospun fibrils confirmed that the fibrils are composed of GelMA (Figure S3). Scanning electron microscope (SEM) analysis showed the smallest variation in fibril diameter with 9% GelMA solution (Figure S4), which was used for subsequent experiments.

We stabilized the electrospun GelMA fibril networks through photo-cross-linking in ethanol solutions containing photoinitiators (Figure 1c). Notably, we observed that if the fibril networks were detached from the aluminum foil and exposed to ethanol solutions containing trace amounts of water prior to photo-cross-linking, they spontaneously shrank and crimped—a phenomenon not previously reported. Previous studies either framed the networks after electrospinning or during photo-cross-linking, likely to address network stability,^36^ or did not provide detailed information on how the networks were handled during these stages.^37^ While these studies indicated that networks dissolved in ethanol solutions with more than 10% water and that photo-cross-linking failed in pure ethanol, they did not specifically address shrinkage in unframed networks. In contrast, our study demonstrates that unframed networks exposed to ethanol solutions of 91%, 93%, 95%, 97%, and pure ethanol exhibited spontaneous shrinkage at varying rates, depending on the ethanol concentration (Figure 2a and Video SM1). This shrinkage was not observed in networks that remained attached to the aluminum foil (Figure 1c) or in those detached from the foil and left in air prior to photo-cross-linking, indicating that shrinkage occurred only in unsecured networks exposed to ethanol solutions with limited water content. The network immersed in 91% ethanol solution shrank the fastest, whereas the one in pure ethanol exhibited minimal shrinkage. The networks in 93–97% ethanol solutions showed intermediate rates of shrinkage, which was asymmetrical and primarily oriented along the direction of fibril alignment. When immersed in 75% and 85% ethanol solutions, the fibrils maintained the fibrillar structure but merged and aggregated into thicker strands in some areas (Figure S5). Immersing the network in ethanol solutions with lower concentrations (such as 50%) led to immediate dissolution. Figure 2b depicts the temporal profiles of network shrinkage versus soaking time. The area of each individual network was measured from the photographs and normalized by the initial area. The shrinkage rate and extent increased as the ethanol concentrations decreased. When immersed in 91% and 93% ethanol solutions, the network rapidly shrank to approximately 15% of its original size within 1 min. The shrinkage rate for the network in 95% ethanol solution was moderate and appeared biphasic, with an initial 30% reduction at a medium rate within the first 3 min, followed by a gradual 30% shrinkage over the next 22 min. Networks immersed in higher-concentration ethanol solutions shrank more slowly and less significantly, reducing to 85.16% and 94.94% of their original size in 97% ethanol solution and pure ethanol, respectively, after 25 min of soaking. These results suggest that the rate and extent of network shrinkage increase with the water content in the ethanol solutions, up to a critical point where the networks dissolve completely. To preserve fibril morphology and enhance post photo-cross-linking stability, we removed excess water and employed critical point drying (CPD), using absolute ethanol as an intermediate solvent (Figure 1c). The fibril morphology was then examined by SEM.

**Figure 2:**
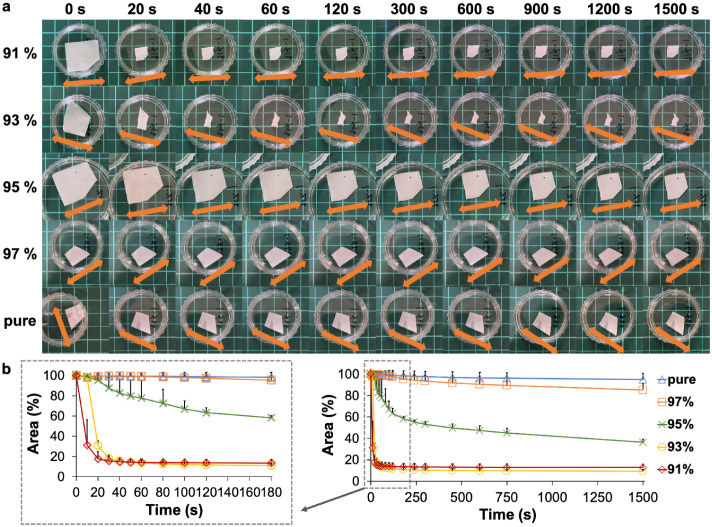
Spontaneous fibril network shrinkage upon limited hydration. (a) Sequential photos of as-spun fibril networks soaked in ethanol solutions of 91%, 93%, 95%, 97%, and pure ethanol, taken at varying intervals over 25 min (square scale: 1 cm). Arrows indicate the direction of fibril alignment. (b) Temporal dynamics of network shrinkage represented by the normalized projected area of the fibril network versus soaking time in ethanol solutions of varying concentrations. (*n* = 3) The adjacent zoom-in plot highlights the first 3 min of temporal dynamics marked by a dotted box.

SEM revealed that the exposure to ethanol solutions containing limited water induced fibril crimping and diameter alterations. Photo-cross-linked fibrils in network attached to the foil and not soaked in ethanol solutions prior to photo-cross-linking exhibited a straight morphology (Figure 3f). In contrast, the fibrils soaked in ethanol solutions of different concentrations exhibited varying degrees of crimping (Figure 3a–e). The crimping degree of individual fibrils is defined as the ratio of the straight distance between the end points of the fibril to its contour length, where a smaller crimping degree corresponds to a greater crimping extent. As shown in Figure 3i, the crimping degrees of the fibrils soaked in ethanol solutions of 91%, 93%, 95%, 97%, and pure ethanol were 0.95 ± 0.05, 0.92 ± 0.06, 0.88 ± 0.10, 0.94 ± 0.08, and 0.94 ± 0.06, respectively (*n* > 50). These values were significantly smaller than those of the straight fibrils, which were estimated to be 0.99 ± 0.00 (*n* > 20), indicating more extensive crimping. Statistical analysis unveiled that soaking fibrils in 95% ethanol solution induced the most pronounced crimping. Fibrils soaked in 95% ethanol solution also frequently exhibited a large-scale, uniformly aligned crimping pattern (Figure 3g,h), with a crimping wavelength of approximately 39.5 ± 7.79 μm (*n* = 208 ridges from 2 fibril networks). The diameters of the fibrils were similar to those of straight fibrils when immersed in 97% ethanol solution and pure ethanol but significantly increased when immersed in ethanol with higher water content (91–95%, Figure 3j). Given that the crimping degree of fibrils immersed in 95% ethanol solution was the most prominent, we chose this ethanol concentration for subsequent experiments. When immersed in water for 24 h, photo-cross-linked electrospun GelMA fibrils with crimping induced by 95% ethanol solution treatment swelled significantly less than the photo-cross-linked GelMA hydrogel chunk, indicating that the fibrillar network is stiffer than the homogeneous structure of the hydrogel (Figure S6).

**Figure 3:**
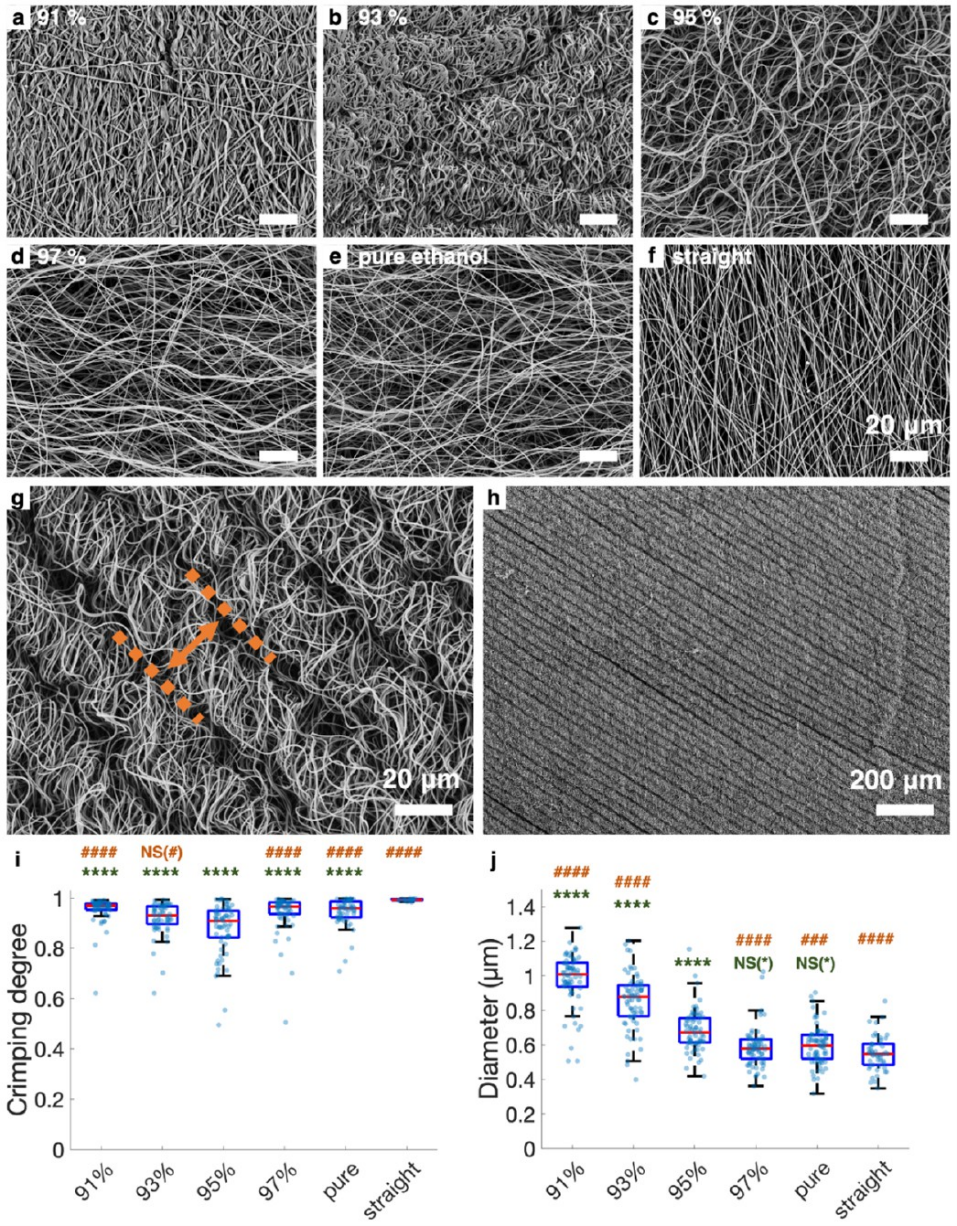
Spontaneous crimping of GelMA fibrils upon limited hydration. SEM images of fibrils soaked in ethanol at concentrations of (a) 91%, (b) 93%, (c) 95%, (d) 97%, and (e) pure ethanol, along with (f) untreated straight fibrils. Images (g) and (h) show the larger-scale, periodic crimping pattern in fibrils soaked in 95% ethanol, with dotted lines marking the edges of the crimp pattern. The arrow indicates the crimping wavelength, defined as the distance between adjacent edges. (i) Crimping degrees and (j) diameters of fibrils treated under various conditions. Comparisons with straight fibrils are marked with *, and comparisons with fibrils soaked in 95% ethanol are marked with #. *P*-values less than 0.05, 0.01, 0.001, and 0.0001 are indicated by *, **, ***, and ****, respectively. The notations NS­(*) and NS(#) indicate that the comparison with straight fibrils and fibrils soaked in 95% ethanol, respectively, was not statistically significant. (*n* = 20–65).

Figure 4 demonstrates the effects of varying soaking durations on the diameters and crimping degrees of fibrils immersed in 95% ethanol. As the soaking duration increased, the fibril diameter gradually increased, while the crimping degree progressively decreased. Both parameters stabilized after approximately 10 min of soaking.

**Figure 4:**
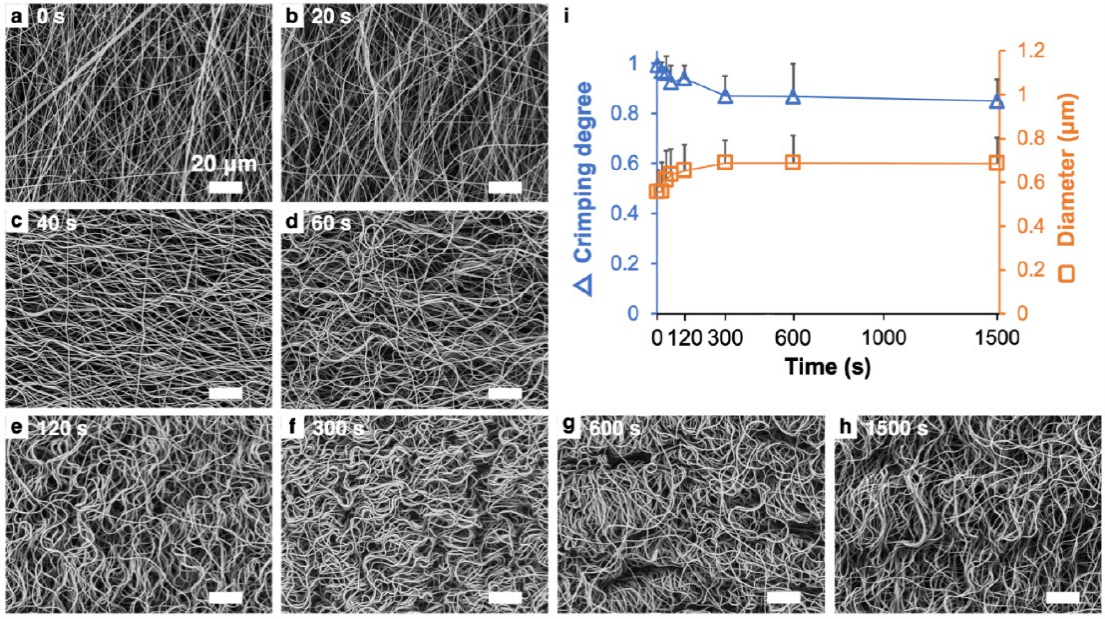
Time-dependent crimping and diameter changes of fibrils under limited hydration. SEM images of fibrils immediately after electrospinning (a) and following immersion in 95% ethanol for (b) 20 s, (c) 40 s, (d) 60 s, (e) 120 s, (f) 300 s, (g) 600 s, and (h) 1500 s. (i) Quantitative analysis of fibril diameters and crimping degrees as a function of soaking time (*n* = 50–70).

### Fibril Crimping Reduced Stiffness and Enhanced Stretchability of Fibril Network

Figure 5 presents representative images and stress–strain profiles for tensile stress analysis of the fibril networks. The networks were cyclically stretched to approximately 1% strain three times for preconditioning using the setup shown in Figure 5a, followed by stretching to failure in the fourth cycle. The preconditioning ensured repeatable stress–strain results and allowed for rigorous comparison of the mechanical properties across various samples. Figure 5b depicts the recorded stress–strain profiles. Young’s modulus and yield point were defined by the slope of the linear elastic region and the end of the elastic region of the stress–strain curve, respectively, while ultimate strength was indicated by the maximum stress during stretching to failure. Note that the stress–strain curves of the four cycles nearly overlapped within the 1% strain range, as highlighted in the dotted box, indicating that the strain applied for preconditioning fell within the elastic region of the materials. This was supported by the SEM images of three samples taken from the same crimped fibril network: before stretching (Figure 5c1), after preconditioning (Figure 5c2), and after stretching to failure (Figure 5c3). The crimped pattern was retained after preconditioning but disappeared after stretching to failure, confirming that preconditioning occurred within the elastic region while stretching to failure caused plastic deformation. Figure 5d displays a schematic diagram illustrating the sequential appearance of a network as it was stretched to failure, along with SEM images corresponding to various parts of a stretched-to-failure crimped fibril network. The images were taken sequentially from the broken end to the clamped end of the network. The crimped pattern was nearly straightened in the parts close to the broken end (Figure 5d1–2), but remained intact near the clamped end (Figure 5d3–4).

**Figure 5:**
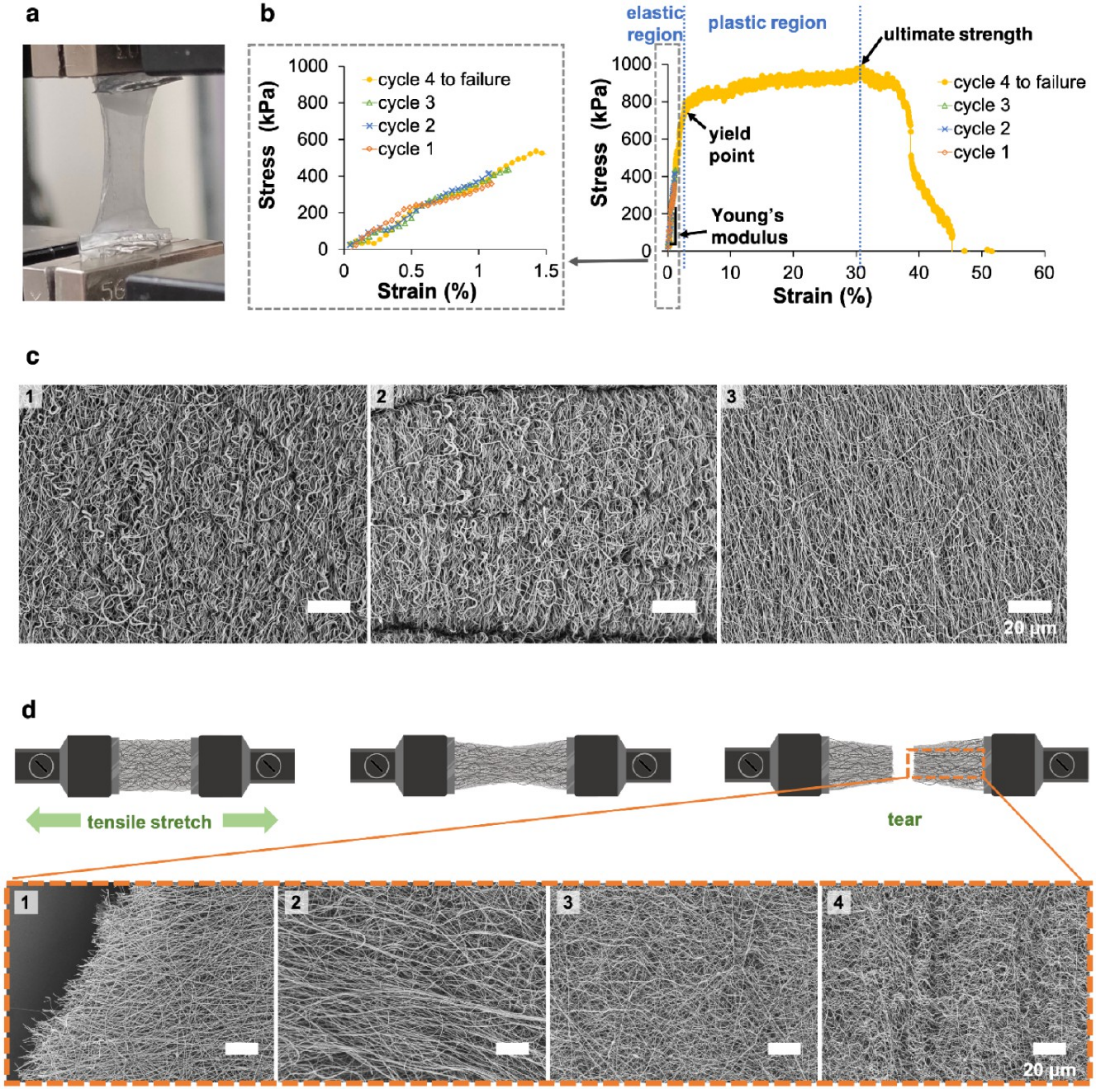
Representative images and stress–strain profiles for tensile stress analysis of fibril network. (a) Photograph of a network clamped for stretching. (b) Stress–strain curves recorded during the four stretching cycles: three for preconditioning and one until failure. The yield point marks the transition from elastic to plastic deformation. Young’s modulus was derived from the initial linear elastic region, and ultimate strength from maximum stress during failure. Stress–strain behavior within the initial 1.5% strain is highlighted (dotted box). (c) SEM images of crimped fibrils: (1) in the unstretched state, (2) after preconditioning, and (3) after stretched-to-failure. (d) Schematic representation of a network stretched to failure, with SEM images taken sequentially from (1) broken end, (2) near broken end, (3) intermediate portion, to (4) clamped end of a stretched-to-failure network.

Figure 6 compares the mechanical properties of various fibril networks analyzed from the stretched-to-failure curves. Figure 6a depicts the stress–strain relationship for parallel-aligned fibril networks under three conditions: as-spun fibrils, photo-cross-linked straight fibrils, and photo-cross-linked crimped fibrils. The solid line connecting symbols represents the average stress measured at each strain point, while the vertical error bars indicate the standard deviation of the stress values. The transition from the elastic to plastic region for these networks is highlighted in the dotted box. Figure 6b shows the stress–strain relationship for as-spun and photo-cross-linked crimped fibrils, both randomly oriented. The data shown in Figure 6a,b were measured from 3 to 5 dried samples for each condition. The measurement for randomly oriented straight fibrils was not provided due to the challenge of maintaining structural integrity when separating the thin network from aluminum foil after photo-cross-linking. Figures 6c–g illustrate a statistical comparison of mechanical properties for these networks. In general, parallel-aligned fibrils exhibited higher average values of Young’s modulus, yield stress, and ultimate stress compared to their randomly oriented counterparts. Parallel-aligned straight fibrils exhibited the highest averaged yield stress of 1.26 MPa (Figure 6c), an average ultimate stress of 1.47 MPa (Figure 6d), and an average Young’s modulus of 43.90 MPa (Figure 6e). Crimped fibrils endured higher elongation at break regardless of fiber orientation. The yield strain and ultimate strain for parallel-aligned crimped fibrils were 4.67% (Figure 6f) and 65.27% (Figure 6g) on average, respectively, both significantly higher than those of parallel-aligned straight fibrils (yield strain of 2.63% and ultimate strain of 10.29% on average). However, the average Young’s modulus for parallel-aligned crimped fibrils was 10.48 MPa, significantly lower than that of straight fibrils (Figure 6e). Additionally, the crimped fibrils exhibited a relatively low yield stress of 484 kPa (Figure 6c) and an ultimate stress of 1.033 MPa on average (Figure 6d). These data substantiate that fibril crimping contributes to a significant decrease in network stiffness and stress, while providing the highest yield and ultimate strain values. This characteristic enhances the network’s ability to endure stretching, protecting the material from easy failure or rupture, thus indicating that such fibril topology resulted in a trade-off between mat’s flexibility and mechanical strength. Figure 6h presents the stress–strain relationship measured from 4 hydrated, crimped fibril networks. The hydrated samples exhibited an average Young’s modulus of 23.27 kPa and a yield stress of 47.29 kPa, both markedly lower than those of the dried fibrils. However, the yield strain of the hydrated fibrils was 163.21% on average, significantly higher than that of the dried ones. This suggests that the material becomes more stretchable in hydrated environments, such as in cell culture or *in vivo* settings.

**Figure 6:**
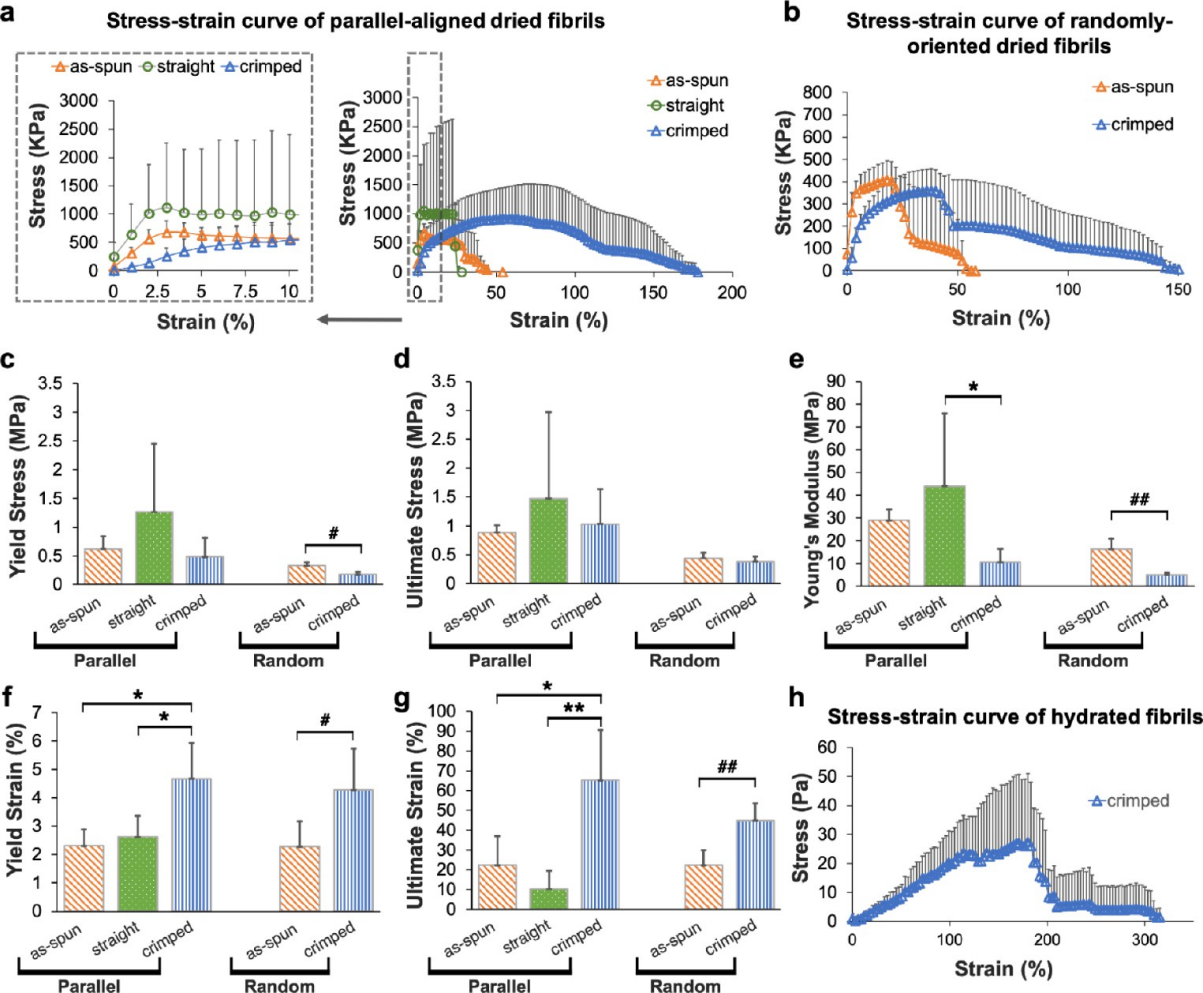
Mechanical properties of various fibril networks. Stress–strain relationship for (a) parallel-aligned and (b) randomly oriented dried fibrils. Statistical comparison for (c) yield stress, (d) ultimate stress, (e) Young’s modulus, (f) yield strain, and (g) ultimate strain between dried fibril networks prepared in various conditions (*n* = 3–5). The marks * denote comparisons within parallel-aligned fibrils, and # denote comparisons within randomly oriented fibrils. *P*-values lower than 0.05, 0.01, 0.001, and 0.0001 are marked as */#, **/##, ***/###, and ****/####, respectively. (h) Stress–strain relationship for hydrated fibrils (*n* = 4).

### Fibril Topology Modulated Cell Phenotypes

After constructing the fibril networks, we conducted *in vitro* studies to evaluate their biocompatibility and influence on cell phenotypes. We first confirmed that the fibril networks were not cytotoxic using the cell counting kit-8 assay, and there was no significant difference in viability between cells cultured on the fibril networks and Petri dishes (Figure S7). We then examined the impact of fibril topology on cell morphology. Analyzing the fluorescent images of NIH 3T3 fibroblasts stained for F-actin and nuclei (Figure 7a) revealed that cells cultured on both straight and crimped fibrils exhibited aligned orientations (Figure 7b). The cytoplasm and nucleus were significantly more elongated in fibroblasts cultured on the fibril networks compared to those on Petri dishes (Figure 7c,e). Cell area was significantly influenced by the topology of the culture materials, being largest on Petri dishes, intermediate on straight fibrils, and smallest on crimped fibrils. Similarly, fibroblasts cultured on the dishes exhibited the largest nuclear area (Figure 7f).

**Figure 7:**
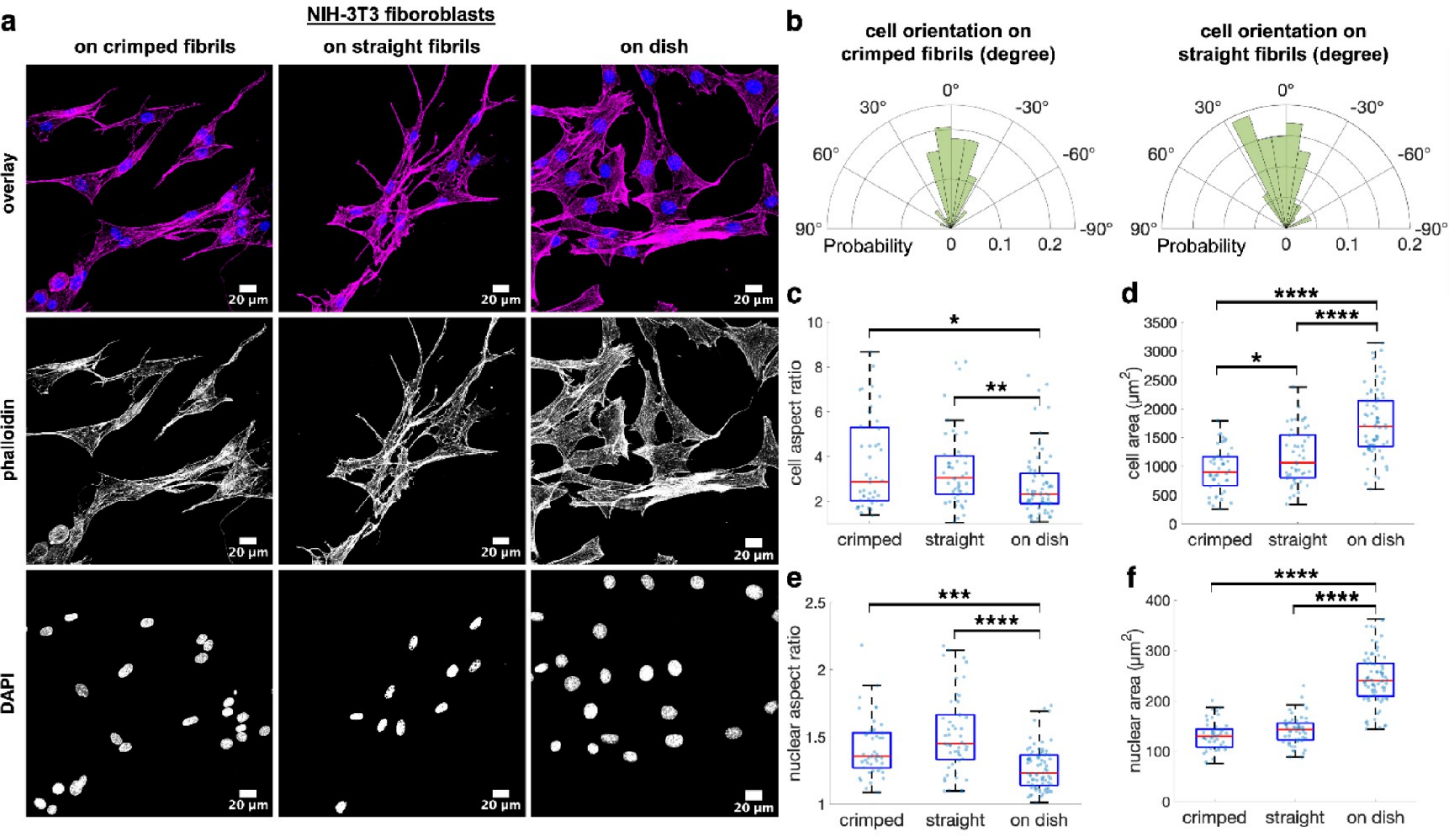
Impact of fibril topology on cell morphology. (a) Representative fluorescence images of NIH-3T3 fibroblasts cultured on various fibril networks and Petri dishes, with F-actin stained using phalloidin (magenta) and nuclei stained using DAPI (blue). (b) Distribution of fibroblast orientation on fibril networks of different topologies, shown by the deviation angles of individual cell orientation from the mean cell orientation. (c) Aspect ratio and (d) area of fibroblasts cultured on various materials, determined from F-actin fluorescence. (e) Aspect ratio and (f) area of cell nuclei, calculated using nuclear fluorescence. *P*-values less than 0.05, 0.01, 0.001, and 0.0001 are denoted as *, **, ***, and ****, respectively, (*n* = 44–76).

Figure 8 illustrates the spatial relationship between cultured cells and fibrils, as well as the effects of cell culturing on fibril topology. Both 3T3 fibroblasts and BMSCs cultured on straight fibrils exhibited a flattened, spreading morphology with extended cellular processes aligned along the fibrils, and remained on the surface of the mat without evidence of infiltration (Figures 8a,b and S8a). In contrast, cells cultured on crimped fibrils showed a less expanded configuration and were frequently covered or wrapped by the fibrils (Figures 8a,b and S8b), suggesting that the cells actively pulled the crimped fibrils toward their bodies, aiding in their translocation and integration within the fibril network, mimicking behavior in a three-dimensional environment. The crimping degrees of fibrils adjacent to adhered fibroblasts and BMSCs in crimped fibril networks remained significantly smaller than those in straight fibril networks after 48 h of culture (Figure 8c,d). Additionally, the crimping degrees of straight and crimped fibril networks after 48 h of cell culture were 0.99 ± 0.01, 0.90 ± 0.07, respectively, which were not significantly different from those observed before cell culture (Figure 3i). Figure 8e shows a crimped fibril network immersed in cell culture media for 48 h. The crimping degrees of these fibrils were not significantly different from those in the networks cultured with fibroblasts and BMSCs for 48 h (Figure 8f). The morphological differences shown in Figure 8a,b prompted us to examine cross sections of the crimped fibril mats. As shown in Figure 8g, fibroblasts cultured on crimped fibrils for 48 h were observed at depths of approximately 200 μm below the mat surface, confirming cell translocation into the interior of the fibril matrix. These findings underscore the effectiveness of crimped fibril architecture in promoting cell-matrix integration, which is crucial for tissue engineering applications. Figure 8h shows fibroblasts growing and expanding along crimped fibrils, using an overlay of immunofluorescence and bright-field images to highlight the spatial relationship between the cells and the fibrils. Figure 8i shows fibroblasts cultured on crimped fibril networks for 7 days (168 h), where the cells proliferated and effectively covered the entire networks, demonstrating the suitability of these networks for long-term cell culture. Notably, fibroblasts on straight fibrils exhibited a more stretched and elongated morphology.

**Figure 8:**
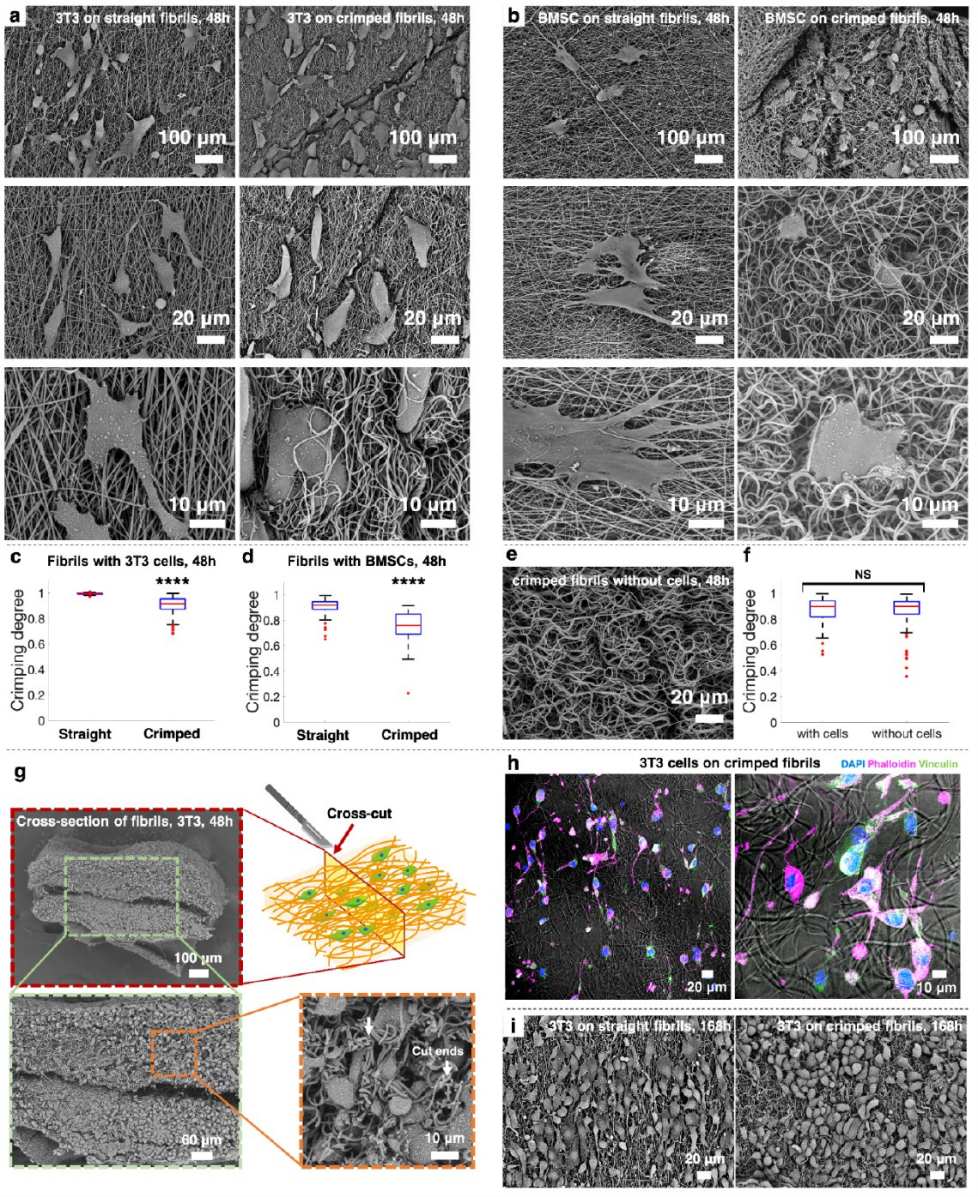
Cellular integration within fibril networks. SEM images of (a) 3T3 fibroblasts and (b) bone marrow-derived mesenchymal stem cells (BMSCs) cultured on straight and crimped fibrils. Statistic comparison of the crimping degree of the fibrils adjacent to adhered (c) fibroblasts and (d) BMSCs between networks with straight and crimped fibrils. (e) Representative SEM image of crimped fibrils soaked in cell culture media without cells for 48 h. (f) Statistic compassion of the crimping degrees of crimped fibrils in networks with and without cell culture. (g) Schematic diagram and SEM images of the cross-section of a crimped fibril mat cultured with fibroblasts for 48 h, showing cell migration to a depth of approximately 200 μm from the mat surface toward the central region. Arrows denote the cut ends of the fibrils at the cross section. (h) Immunofluorescence images of fibroblasts cultured on crimped fibrils, overlaid with bright-field images. (blue for DAPI, magenta for phalloidin, green for vinculin) (i) SEM images of fibroblasts cultured on straight and crimped fibrils for 7 days. Cells had a rounder shape on crimped fibrils, similar to those observed after 48 h culture. *P*-values lower than 0.0001 are marked as ****. (*n* = 50 in panels c, d, f).

We investigated whether culturing cells on fibrils with different topologies induced changes in protein expression. Figure 9a shows representative immunofluorescent images of 3T3 cells grown on straight and crimped fibrils, and Petri dishes for 48 h, stained for F-actin (magenta), nucleus (blue), α-tubulin (yellow), and vinculin (green). Quantitative analysis revealed that culturing fibroblasts on crimped fibrils significantly increased the fluorescence intensity per cell of vinculin and α-tubulin (Figure 9b,d), and the vinculin-positive area per cell (Figures 9c). These results suggest that crimped fibrils may enhance cell-matrix adhesion through increased vinculin expression and facilitate cell translocation by promoting tubulin expression. Figure 9e presents immunofluorescent images of fibroblasts stained for α-smooth muscle actin (α-SMA, yellow) and Yes-associated protein 1 (YAP1, green) after 48 h of culture on Petri dishes or fibrillar structures. The expression of α-SMA was notably lower in cells cultured on fibrils compared to those on Petri dishes, with fibroblasts on straight fibrils exhibiting significantly higher α-SMA intensity than those on crimped fibrils (Figure 9f). This indicates that crimped fibrils reduced the transformation of fibroblasts into myofibroblasts. Finally, culturing cells on either straight or crimped fibrillar structures did not result in significant changes in the nuclear-to-cytoplasmic expression ratio of YAP1 compared to those cultured on Petri dishes (Figure 9g).

**Figure 9:**
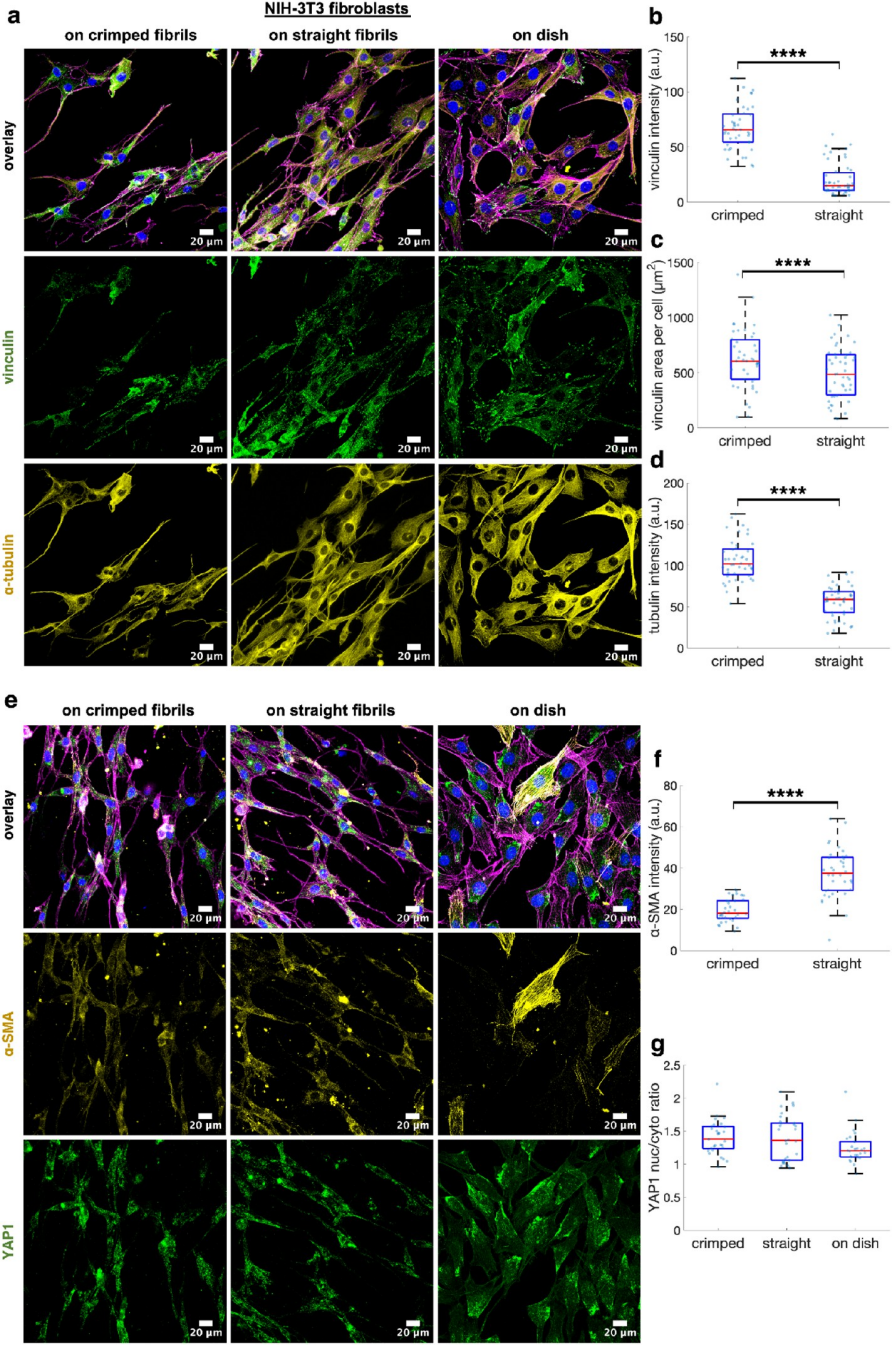
Impact of fibril topology on protein expression. (a) Immunofluorescence images of NIH-3T3 fibroblasts cultured on Petri dishes, straight fibrils, or crimped fibrils for 48 h, stained for F-actin (magenta), nucleus (blue), α-tubulin (yellow), and vinculin (green). Statistical comparison of (b) fluorescence intensity per cell for vinculin expression, (c) vinculin expression area per cell, and (d) fluorescence intensity per cell for α-tubulin expression. (e) Immunofluorescence images of fibroblasts cultured on Petri dishes or fibrillar structures for 48 h, stained for F-actin (magenta), nucleus (blue), α-SMA (yellow), and YAP1 (green). Statistical comparison of fluorescence intensity for (f) α-SMA and (g) YAP1 expression. Note that α-SMA data for cells cultured on Petri dishes were not included in the comparison due to different fluorescence settings used for image acquisition. *P*-values less than 0.0001 are denoted as **** (*n* = 26–36 cells in panels b, c; *n* = 44–47 cells in panels e, f).

## Discussion

Our work introduces a novel finding. Upon exposure to low concentrations of water, electrospun GelMA fibrils developed a self-induced crimped configuration, and the entire fibril network shrank. Although GelMA fibrils without photo-cross-linking were previously thought to dissolve in most aqueous environments, our results demonstrate that under limited water content, these fibrils not only retain their structural integrity—consistent with Bridge et al.^36^—but also form a crimped pattern. We hypothesize that this crimping arises from restricted hydrogen bond formation within and between fibrils in a weakly aqueous environment. Although both ethanol and water can participate in hydrogen bonding, water generally forms stronger and more extensive hydrogen bond networks. This is attributed to its higher dielectric constant,^38^ higher dipole moment,^39^ and greater number of hydrogen bond donors and acceptors compared to ethanol.^40^ Moreover, prior studies have shown that water molecules contribute more effectively to atomic connectivity than ethanol in mixed-solvent systems, most likely due to the steric hindrance of the ethyl group in ethanol.^41^ These differences suggest that, while ethanol plays a role, water is likely the dominant contributor to the hydrogen bonding interactions that influence the crimping behavior of GelMA fibrils. During electrospinning, non-cross-linked GelMA molecules are deposited into distinct fibrils (Figure 10a). When exposed to ethanol solutions containing at least 95% ethanol, the limited absorption of water molecules leads to uneven formation of intra- and interfibril hydrogen bonds, causing the fibrils to buckle into a wavy pattern (Figure 10b). As water content increases, the absorption of additional water molecules promotes more uniform hydrogen bonding, which causes the fibrils to swell and promotes their aggregation (Figure 10c). At higher water content (ethanol concentration below 50%), the fibrils absorb enough water to dissolve the GelMA chains entirely (Figure 10d). However, the validity of this hypothesis remains uncertain and warrants further investigation. After UV cross-linking and critical point drying (CPD), the crimped architecture is preserved, and the fibrils become water-insoluble. We confirmed the stability of these crimped structures by demonstrating that they remained intact, with no significant change in crimping degree, after 48 h in cell culture medium, ensuring stability for extended cell culture.

**Figure 10:**
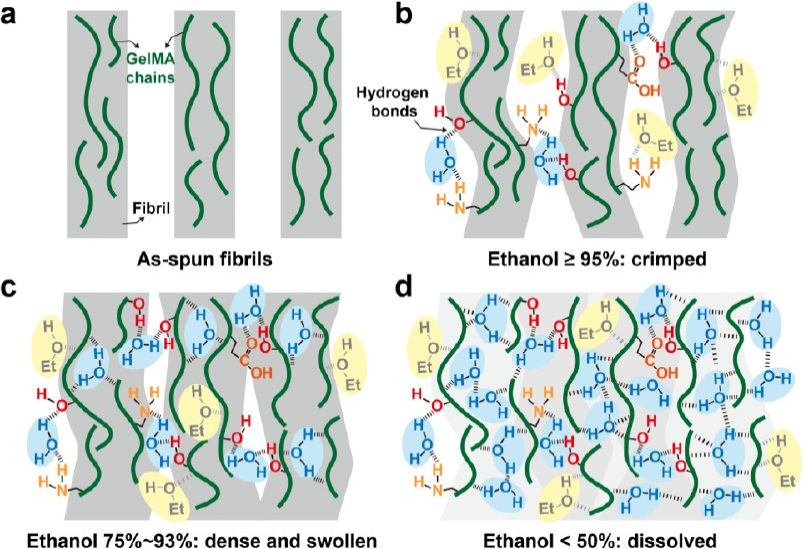
Proposed mechanisms of ethanol concentration-dependent topology changes in GelMA fibrils induced by limited hydration. (a) Non-cross-linked GelMA chains confined within as-spun fibrils. (b) Fibrils become buckled due to interactions with hydrogen bonds provided by water and ethanol molecules when immersed in high-concentration ethanol. (c) Increased water content promotes more hydrogen-bonding network inside the fibrils, resulting in fibril thickening and aggregation. (d) Fibrils completely dissolve when the ethanol concentration drops below 50%.

Our innovative constructs provide an *in vitro* platform mirroring the topology of young native tissues, promising for elucidating the intricate mechanisms governing 3D cell-matrix interactions. The smallest diameter of our synthesized GelMA fibrils is approximately 300 nm, matching the upper limit of native collagen fibril diameters (30–300 nm). The wavelength of the large-scale, periodic crimping pattern (Figure 3h) is around 40 μm, within the range observed in native collagen tissues (10–100 μm). Modulating fibril topology balances mechanical strength and flexibility in the constructs. Compared to straight fibrils, crimped fibrils exhibit increased stretchability, enhanced rupture resistance, and reduced stiffness, similar to native tissues. This characteristic allows the fibril network to act as effective shock absorbers, reducing internal stress within the matrix during external stretching—crucial for applications requiring both mechanical resilience and flexibility, such as tissue engineering and regenerative medicine. The observed high yield and ultimate strain values of the crimped fibrils seem to be connected to the shrinkage of the fibril network. After immersion in 95% ethanol solution for 25 min, the fibril networks contracted to an average of about 58% of their initial length along the direction of fibril alignment. The ultimate strain of these networks averaged around 65%, indicating they were stretched to approximately 1.65 times their length at the start of stretching. Notably, multiplying 0.58 by 1.65 yields a value close to 1 (0.957), suggesting that the stretching nearly restored the network to its original precrimped length. This correlation implies that the high ultimate strain values may be largely influenced by the initial network shrinkage. Our fibril networks were noncytotoxic and support favorable cell attachment. Cells cultured on the fibril networks exhibited a spindle-like, aligned morphology, highlighting the supportive growth environment and adhesive properties of the fibrils. The crimped fibril architecture effectively promoted cell translocation into the central region of the 3D network, suggesting that the crimp-induced porous microenvironments facilitate cell migration along the fibrils.

Our data indicate that the mechanical and topological characteristics of the fibrillar structure significantly affected the phenotypes of resident cells. Cells attached to straight fibrils exhibited a more flattened, spreading morphology, larger cell area, higher expression level of α-SMA, and lower expression level of vinculin and α-tubulin, compared to those anchored to crimped fibrils. The difference in cellular morphology may be attributed to the relatively planar, two-dimensional (2D) surface provided by the straight fibrils for cellular engagement. The reduced cell spread on crimped fibrils is consistent with findings by Davidson et al.^26^ Similarly, the increased α-SMA expression in cells cultured on straight fibrils aligns with the general observation that stiffer surfaces promote α-SMA expression.^42^ However, the mechanisms behind the increased vinculin and α-tubulin expression in cells cultured on crimped fibrils remain unclear and warrant further investigation. Typically, stiffer substrates enhance vinculin expression,^26,43^ and YAP1 nuclear translocation,^44^ which does not account for our findings, as the crimped fibril network is softer than the straight one. This discrepancy may stem from the differences in topology between the fibril networks. The straight fibril network predominantly provides 1D and 2D-like topologies, which have been shown to facilitate long-range mechanical force transmission and consequently elicit cellular responses commonly observed in rigid environments in previous studies.^45^ In contrast, the crimped fibril network offers a more 3D-like topology, where mechanotransduction is more complex due to uneven distribution of cell-matrix adhesions.

Our study has several limitations. We did not comprehensively investigate the mechanical properties of hydrated fibrils due to their slippery nature. Understanding these properties could provide insights into the performance of fibrils in physiological environments and the interactions between cells and fibrillar patterns. The integration of cells within the fibril network was examined using SEM images, which were limited to the network surface. Using confocal and/or multiphoton microscopy to examine fluorescence-labeled fibrils and cells could provide valuable insights into their spatial relationships within the network. Additionally, our soaking conditions were limited to ethanol solutions; future studies could explore other organic and ionic solutions. Finally, most of our results were derived from NIH 3T3 fibroblasts and BMSCs, and further studies are needed to determine the platform’s applicability to other cell types.

In summary, we present a novel and straightforward method for generating crimped nanofibrils from collagen-derived materials. The resulting construct is biocompatible and closely mimics native tissues in both topological and mechanical properties, facilitating cell translocation and integration. The crimping of the fibrils demonstrates high adjustability, enabling tailored patterning and functionality. This is especially valuable for studying the interactions between cell behavior and matrix topology. Our work holds significant promise for *in vitro* platforms designed to explore cell-matrix dynamics and has the potential to be applied across various scientific and medical fields, including drug delivery systems and tissue engineering scaffolds.

## Supplementary Material





## Data Availability

The data that support the findings of this study are available from the web link: Kuo, Po-Ling (2024), “Spontaneous crimping of GelMA fibrils”, Mendeley Data, V1, doi: 10.17632/8wjx4hvn3b.1.

## Notes

Spontaneous fibril network shrinkage upon limited hydration (MP4)
SEM images of GelMA fibrils with and without washing for LAP, ^1^H NMR spectra of type-A gelatin and GelMA, ^1^H NMR spectra of electrospun GelMA fibrils, effects of GelMA concentrations on electrospun fibril diameter, SEM images of fibrils soaked in 75% and 85% ethanol, temporal profile of the swelling ratio for photo-cross-linked GelMA hydrogels and photo-cross-linked electrospun fibril networks with crimping induced by 95% ethanol, cell viability for GelMA fibril networks, SEM comparison of cellular localization on straight and crimped fibrils, and degree of MA substitution determined by ^1^H NMR and TNBS assay (PDF)
