# Supplementary material for: Spontaneous Crimping of Gelatin Methacryloyl Nanofibrils Induced by Limited Hydration
Source: ACS Biomater Sci Eng. 2025 Jul 18;11(8):4758–72. doi: 10.1021/acsbiomaterials.5c00828 (PMC12344688; doi:10.1021/acsbiomaterials.5c00828)
Supplement: Supplementary file 2 [file ab5c00828_si_002.pdf]

Supplementary Information

Spontaneous Crimping of Gelatin Methacryloyl Nanofibrils Induced by Limited Hydration

Chien-Wei Wu[1,2,3], Tsu-Yin Huang[1], Liang-Jie Huang[1], Yi-Wei Kuo[1], Chin-Lin Guo[3], Po-Ling Kuo[1,2,4,5*]

[1]Graduate Institute of Biomedical Electronics and Bioinformatics, National Taiwan University, No. 1, Sec. 4, Roosevelt Rd., Taipei 106, Taiwan

[2]Department of Electrical Engineering, National Taiwan University, No. 1, Sec. 4, Roosevelt Rd., Taipei 106, Taiwan

[3]Institute of Physics, Academia Sinica, No. 128, Academia Rd. Sec. 2, Taipei 115, Taiwan

[4]Department of Physical Medicine and Rehabilitation, National Taiwan University Hospital, No.1, Chang De St., Taipei 100, Taiwan

[5]College of Medicine, National Taiwan University, No.1, Jen Ai Rd., Sec. 1, Taipei 100, Taiwan

* Corresponding author

Movie SM1. Spontaneous fibril network shrinkage upon limited hydration. Video was taken for as-spun fibril networks soaked in ethanol solutions with concentrations of 91%, 93%, 95%, 97%, and pure ethanol for 25 minutes.

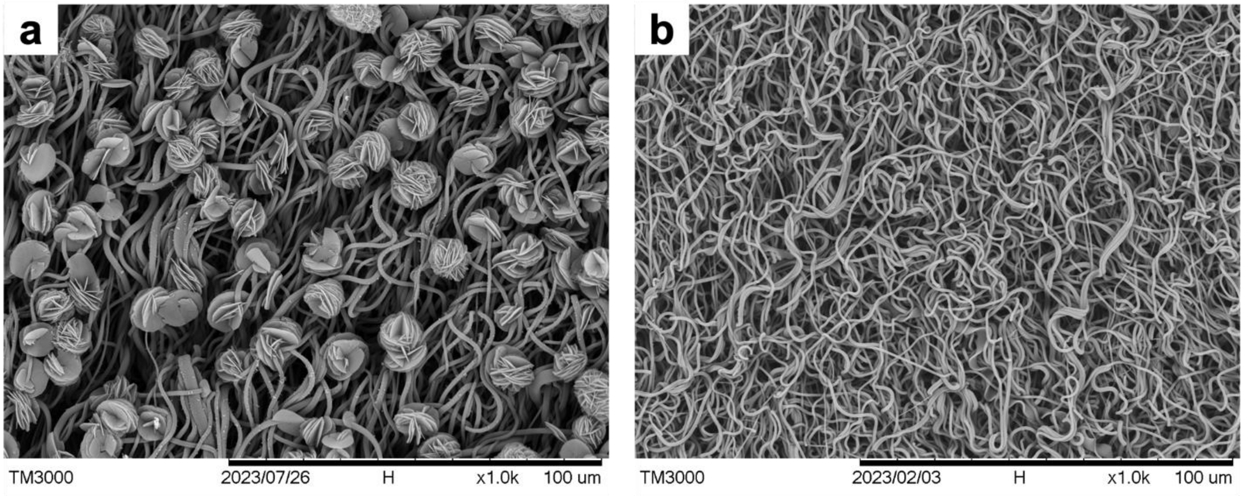

Figure S1 | SEM comparison of unwashed and washed GelMA fibrils showing LAP removal. (a) SEM image of unwashed fibrils reveals the presence of residual LAP within the network, which appears as surface deposits after dehydration. (b) SEM image of washed fibrils shows a clean fibril network with no observable LAP residues, indicating effective removal of the photoinitiator.

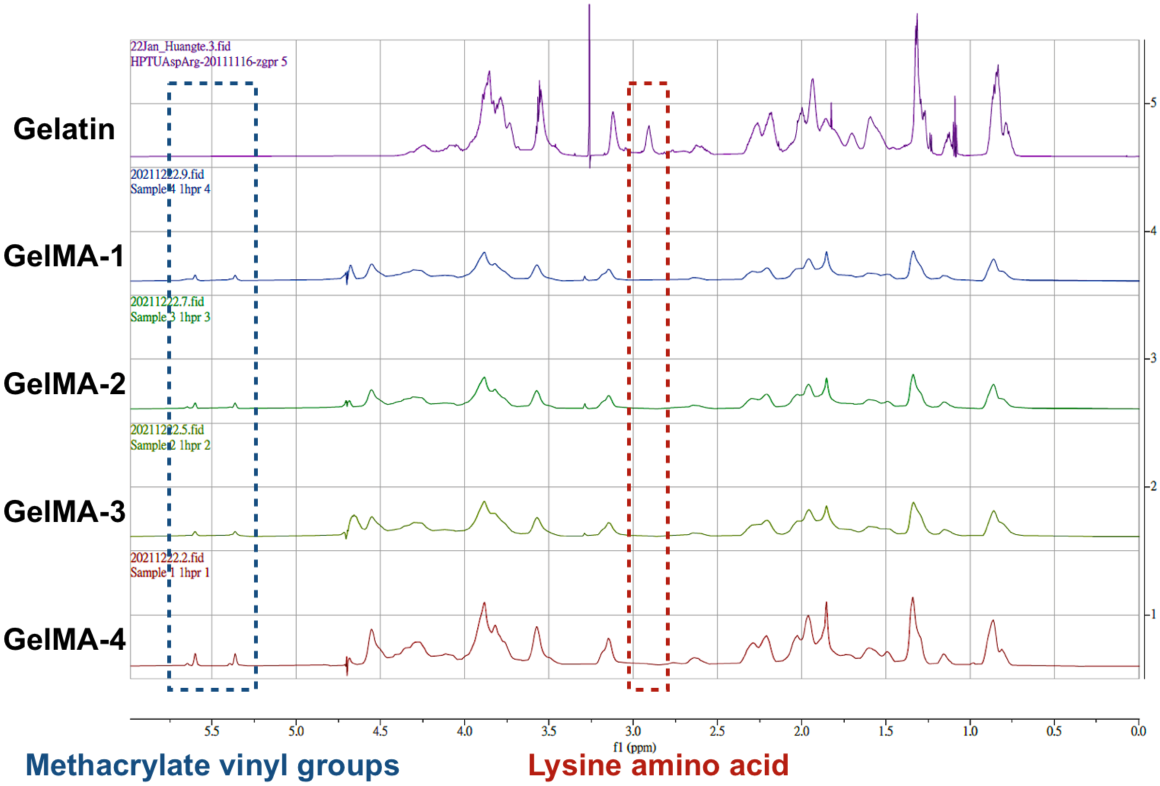

Figure S2 | ^{1}H-NMR spectra of type-A gelatin and four GelMA samples synthesized under identical conditions. Chemical shifts at 5.4–5.5 ppm and 5.6–5.7 ppm, representing the acrylic protons ($CH_2=C(CH_3)CONH$-) in methacrylate vinyl groups, are marked in blue. The decreased signal at 2.85–2.95 ppm, representing the lysine amino acid ($NH_2C\underline{H_2}CH_2CH_2CH_2$) substitution, is marked in red. The successful methacrylate substitution in GelMA is indicated by the new proton peaks at 5.4–5.5 ppm and 5.6–5.7 ppm, and the disappearance of the proton peaks at 2.85–2.95 ppm, representing lysine amino groups. The degree of substitution (DS, %) was calculated by comparing the integral area of the peaks at 2.85–2.95 ppm in type-A gelatin and GelMA.

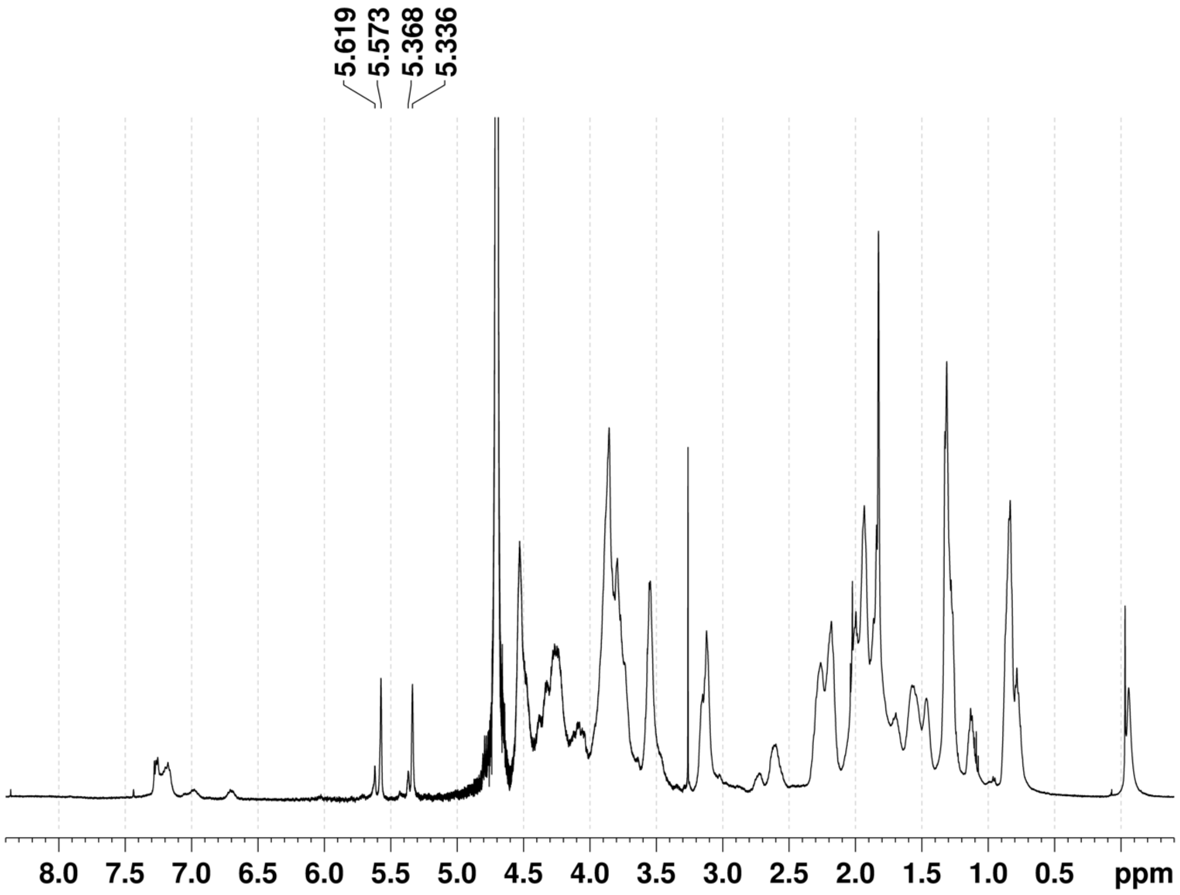

Figure S3 | ^{1}H-NMR spectra of electrospun GelMA fibrils. The chemical shifts at 5.4-5.5 and 5.6-5.7 ppm, representing the acrylic protons (C**H₂**=C(CH₃)CONH-) in methacrylate vinyl groups, confirm that the electrospun fibrils are GelMA.

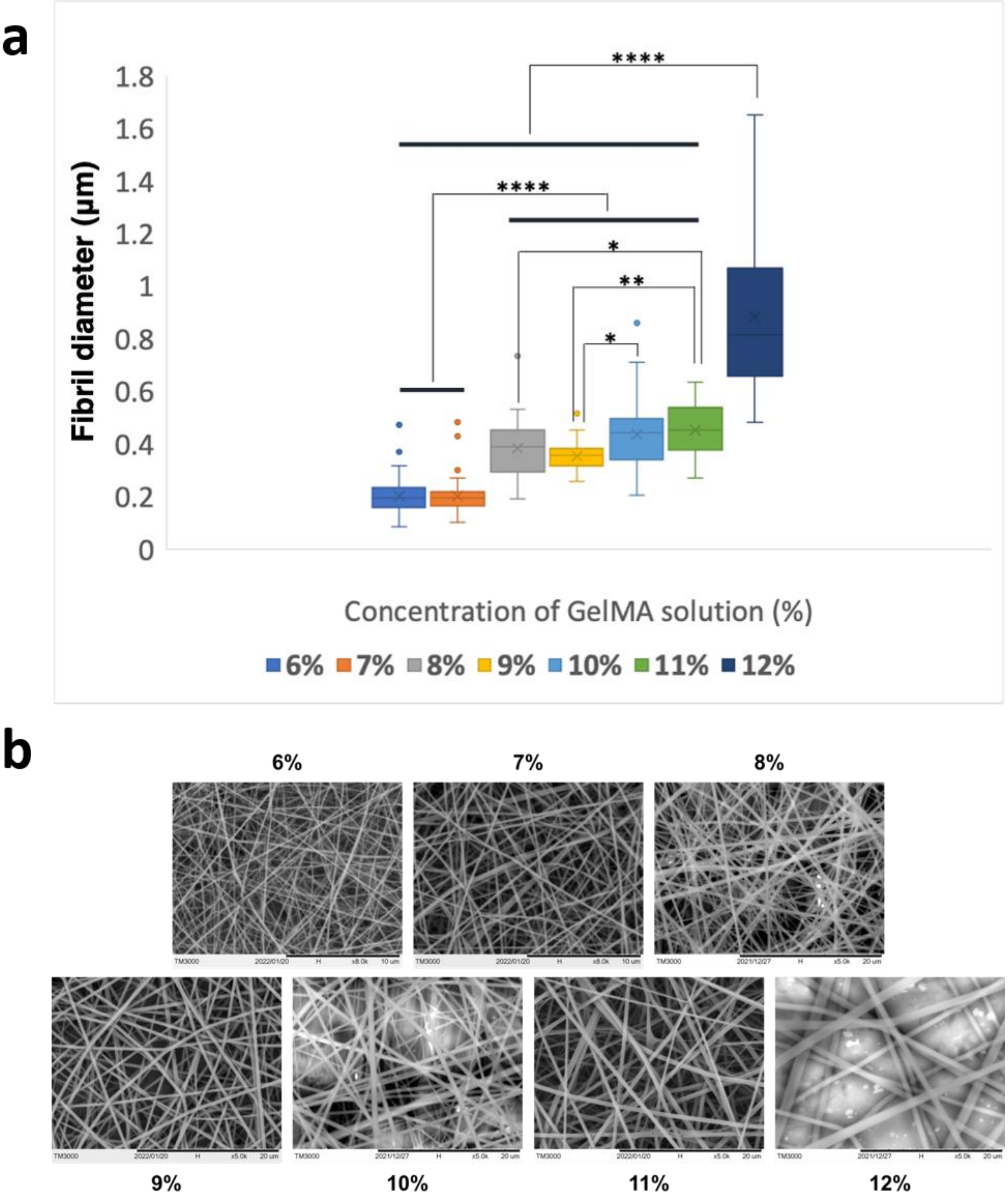

Figure S4 | Effects of GelMA concentrations on electrospun fibril diameter. (a) Variation in fibril diameter generated by GelMA solutions ranging from 6-12% concentrations. Significant differences are marked as *, **, ***, and **** for p-values < 0.05, 0.01, 0.001, and 0.0001, respectively (n=50). Higher

GelMA concentrations produced larger fibril diameters, though the relationship was not linear. Diameters can be grouped by GelMA concentration: 6-7%, 8-9%, 10-11%, and 12%, with significant differences between groups but not within them. The 9% GelMA solution showed the smallest diameter variance. (b) Representative SEM images of fibrils electrospun from different GelMA concentrations.

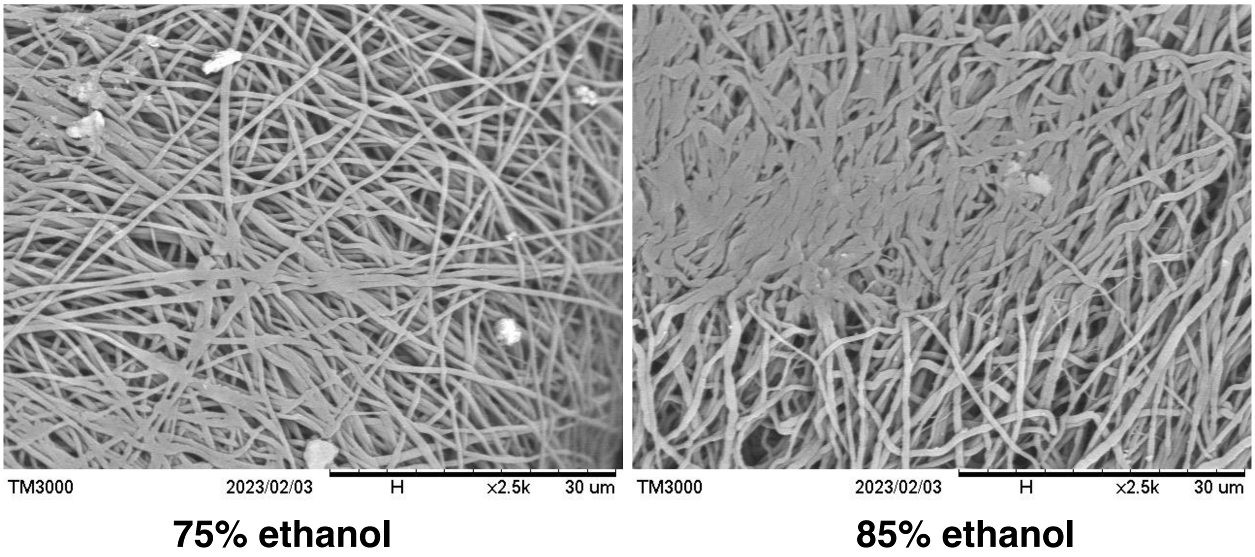

Figure S5 | SEM images of fibrils soaked in 75% and 85% ethanol solutions. Fibrils preserved their fibrillar structure but merged and aggregated into thicker strands in some areas.

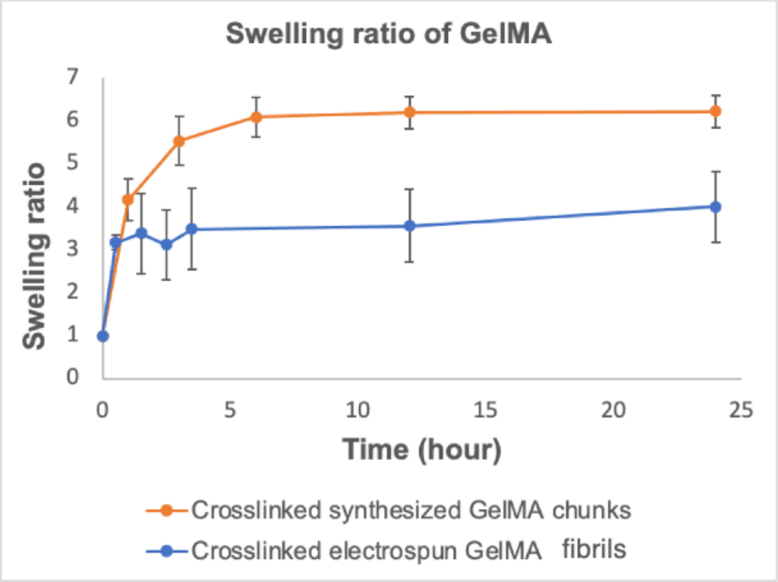

Figure S6 | Temporal profile of the swelling ratio for photocrosslinked GelMA hydrogel chunks (n=10) and photocrosslinked electrospun fibril networks with crimping induced by 95% ethanol treatment (n=5). The swelling ratio stabilized at 6 hours for the chunks and 4 hours for the fibril networks, reaching values of 6.23±0.28 and 4.01±0.83, respectively, after 24 hours of soaking in water.

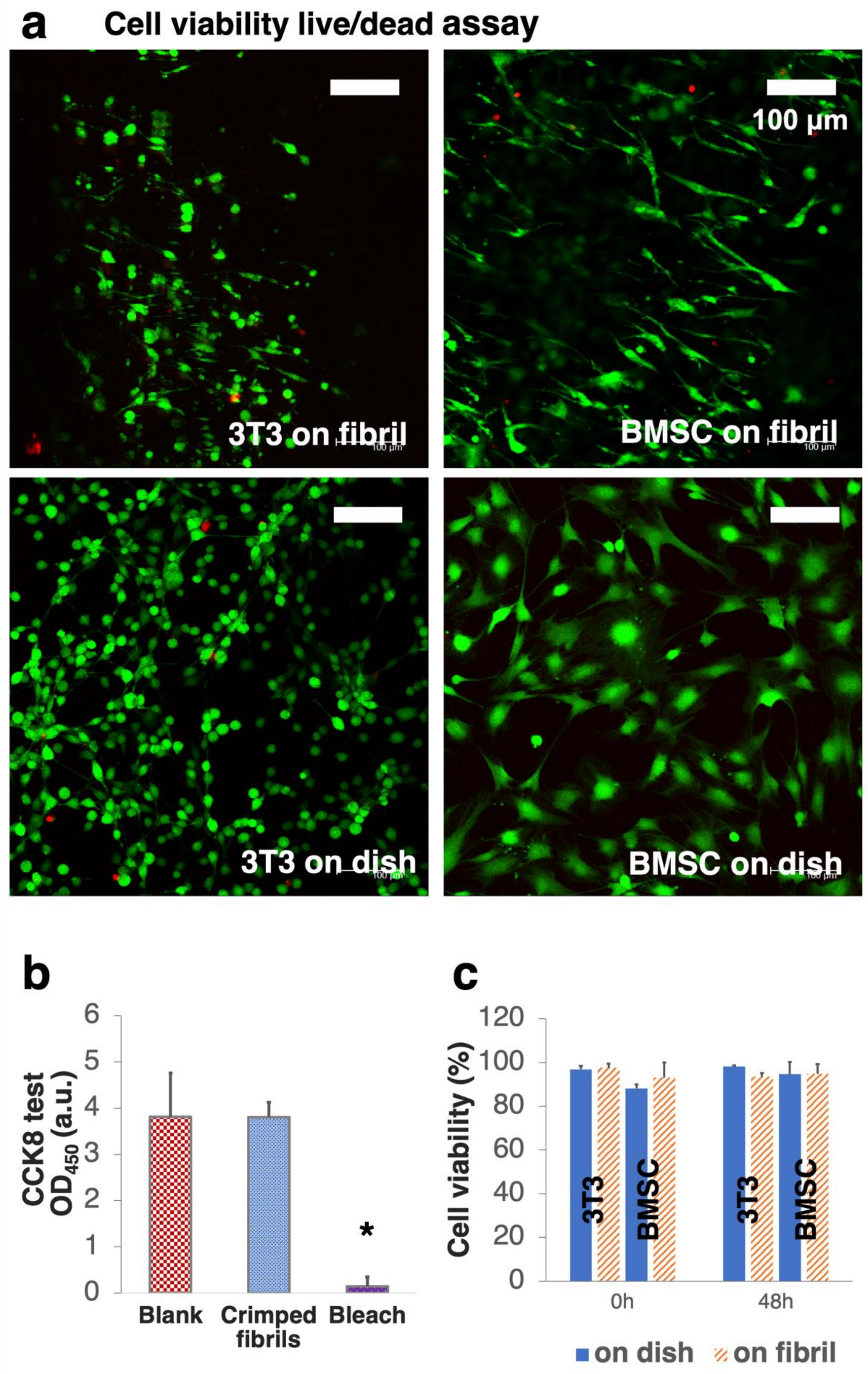
a
Cell viability live/dead assay
100 µm
3T3 on fibril
BMSC on fibril
3T3 on dish
BMSC on dish
b
CCK8 test
OD450 (a.u.)
6
5
4
3
2
1
0
*
Blank
Crimped fibrils
Bleach
c
Cell viability (%)
120
100
80
60
40
20
0
3T3
BMSC
3T3
BMSC
0h
48h
on dish
on fibril

Figure S7 | Cell viability for GelMA fibril networks. (a) Fluorescent images of NIH 3T3 and BMSC cells cultured on crimped fibril mats and Petri dishes for the live/dead assay. Live cells stained with Calcein AM emitted green fluorescence, while dead cells identified by Propidium Iodide fluoresced red. Both cell types exhibited spindle-like morphology and directional orientation on the fibril mats, contrasting with a more randomized orientation on Petri dishes. (b) Cytotoxicity of NIH 3T3 cells cultured in various media quantified by optical density at 450 nm using the cell counting kit-8 assay. "Blank" refers to cells in standard growth medium (positive control, 100% survival), "Crimped fibrils" refers to cells in the presence of crimped fibrils, and "Bleach" refers to cells in medium containing bleach (negative control, 0% survival). Bars and error bars represent mean values and standard deviations. The asterisk (*) indicates a p-value < 0.05 (n=5). Results show no significant difference in cell viability between crimped fibril and Blank conditions. (c) Viability of 3T3 and BMSC cells cultured on different materials for 48 hours. Viability was calculated as the ratio of live cells to total cells. Bars and error bars represent mean values and standard deviations. Statistical analysis using the Kruskal-Wallis Test showed no significant difference in cell viability between cultures on fibril mats and Petri dishes (n=50).

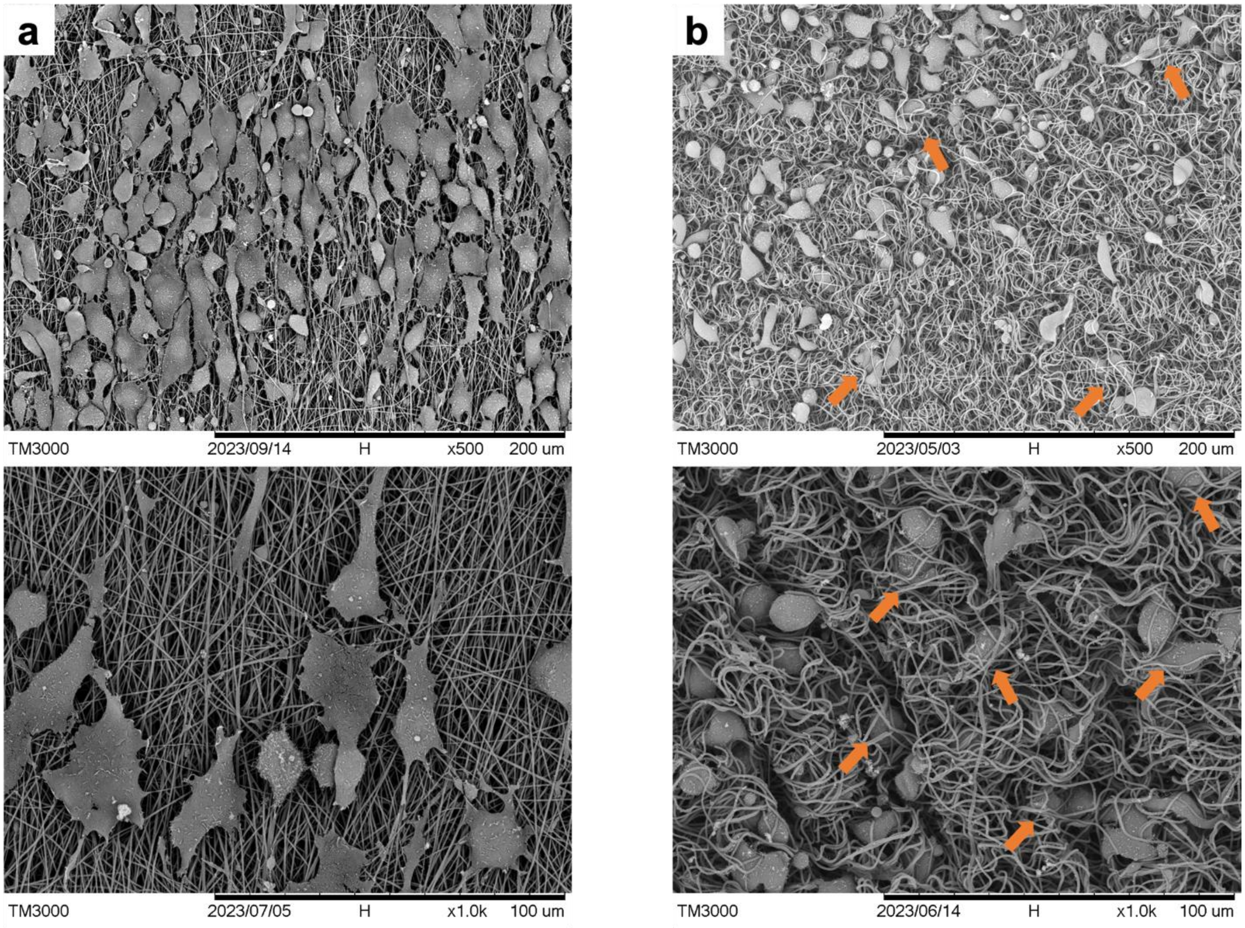

Figure S8 | SEM comparison of cellular localization on straight and crimped fibrils. (a) SEM image showing cells cultured on straight fibrils, exhibiting surface adherence without evidence of infiltration or fibril wrapping. (b) SEM image showing cells cultured on crimped fibrils, with a notable portion of cells partially enveloped or wrapped by the surrounding fibrils (indicated by arrows), suggesting enhanced cellular integration into the fibril network.

Table S1. Degree of MA substitution (DS, %) determined by ^{1}H-NMR and TNBS assay.

GelMA	DS(%) with ^{1}H-NMR assay	DS(%) with TNBS assay
Sample_1	94.46	79.50 ± 3.16
Sample_2	93.60	79.45 ± 5.15
Sample_3	94.16	79.46 ± 4.25
Sample_4	94.32	78.94 ± 7.23
